# The effect of antenatal education in small classes on obstetric and psycho-social outcomes - a systematic review

**DOI:** 10.1186/s13643-015-0010-x

**Published:** 2015-02-28

**Authors:** Carina Sjöberg Brixval, Solveig Forberg Axelsen, Stine Glenstrup Lauemøller, Stig Krøger Andersen, Pernille Due, Vibeke Koushede

**Affiliations:** National Institute of Public Health, University of Southern Denmark, Copenhagen, Denmark

**Keywords:** Antenatal education classes, Obstetric, Labor, Birth, Parenting, Parenthood, Psycho-social, Postnatal depression, Systematic review, Randomized trials

## Abstract

**Background:**

The aims of antenatal education are broad and encompass outcomes related to pregnancy, birth, and parenthood. Both form and content of antenatal education have changed over time without evidence of effects on relevant outcomes. The effect of antenatal education in groups, with participation of a small number of participants, may differ from the effect of other forms of antenatal education due to, for example, group dynamic. The objective of this systematic review is to assess the effects of antenatal education in small groups on obstetric as well as psycho-social outcomes.

**Methods:**

Bibliographic databases (Medline, EMBASE, CENTRAL, CINAHL, Web of Science, and PsycINFO) were searched. We included randomized and quasi-randomized trials irrespective of language, publication year, publication type, and publication status. Only trials carried out in the Western world were considered in this review. Studies were assessed for bias using the Cochrane risk of bias tool. Results are presented as structured summaries of the included trials and as forest plots.

**Results:**

We identified 5,708 records. Of these, 17 studies met inclusion criteria. Studies varied greatly in content of the experimental and control condition. All outcomes were only reported in a single or a few trials, leading to limited or uncertain confidence in effect estimates. Given the heterogeneity in interventions and outcomes and also the high risk of bias of studies, we are unable to draw definitive conclusions as to the impact of small group antenatal education on obstetric and psycho-social outcomes.

**Conclusions:**

Insufficient evidence exists as to whether antenatal education in small classes is effective in regard to obstetric and psycho-social outcomes. We recommend updating this review following the emergence of well-conducted randomized controlled trials with a low risk of bias.

**Systematic review registration:**

PROSPERO CRD42013004319

**Electronic supplementary material:**

The online version of this article (doi:10.1186/s13643-015-0010-x) contains supplementary material, which is available to authorized users.

## Background

Antenatal education is offered to pregnant women in most high-income countries, more recently also to expecting fathers. Antenatal education has the overall aim of providing expecting parents with strategies for dealing with pregnancy, childbirth, and parenthood [1]. More specific aims include increasing knowledge, e.g., on antenatal and postnatal depression, the birth process, pain relief and obstetric interventions, promoting breast feeding, and increasing confidence in women’s ability to give birth as well as becoming parents. Also, information imparted on health promotion and risk reduction is an important aim of antenatal education. Meeting others in the same situation and developing social networks is another aim of antenatal classes [2].

Antenatal education is well-established in many Western countries, but the type and arrangement of the education is debated. Antenatal education has been sensitive to opinions and trends, and both form and content have undergone marked changes over time. During certain periods, practice has been centered on antenatal education in small classes with group discussions - in others, the practice has been lectures in large auditoriums. Also, the content has varied greatly. Topics like, for example, breathing and/or relaxation techniques have been included and left out of antenatal education intermittently. Due to financial and structural changes in the health care sector, the numbers of antenatal education sessions have also changed over time [2]. All these changes have occurred without evidence of an effect of antenatal education on outcomes relevant to expecting parents as well as health care providers [3].

Current evidence points to the importance of interacting with fellow learners and the learning environment in order to obtain new competencies [4]. In antenatal education classes with a small number of participants, it is possible to create an environment which enables expecting parents to discuss feelings and concerns. Furthermore, it may enhance their awareness of their own resources and provide them with problem-solving strategies that enhance important competencies to cope with birth and parenthood [5]. However, this approach has not been subject to thorough scrutiny.

In health care systems with limited resources, policy makers should be able to make informed decisions about health care priorities based on scientific evidence [6]. According to service providers, insecure parents use the health care services beyond indication. Janicke and Finney suggested that the use of pediatric services is a function of perceived parental stress and low self-efficacy related to coping with life demands [7]. Antenatal education in small classes may increase parenting resources leading to health care cost savings in the long term although the immediate expenses are larger for small classes than for auditorium lectures.

A previous systematic review by Gagnon and Sandall from 2007 investigated the effect of structured antenatal education, including antenatal education in small classes, either to individuals or groups on a range of outcomes both related to the birth process and parenthood and concluded that the effect of general antenatal education for childbirth or parenthood or both remains largely unknown [3].

A systematic review is needed in order to assess currently available evidence for the effectiveness of antenatal education in small classes compared to no or other forms of education. The aims of antenatal education are numerous and vary in nature. Therefore, the objective of this systematic review is to assess the effectiveness of antenatal education in small classes on obstetric and psycho-social outcomes compared to standard care or other types of educational programs using randomized trials from Western countries.

## Methods

We carried out this systematic review using the Cochrane Handbook for Systematic Reviews of Interventions as a guide [8]. We published our methods as a protocol before conducting the review [9] and registered the review within the International Prospective Register of Systematic Reviews (PROSPERO) (registration number CRD42013004319 http://www.crd.york.ac.uk/PROSPERO/register_new_review.asp?RecordID=4319&UserID=2668). This systematic review is reported according to the PRISMA statement [10] [see Additional file 1].

### Search strategy

Extensive searches were performed by an information specialist (SKA). The databases Medline, EMBASE, CENTRAL, CINAHL, Web of Science, and PsycINFO were searched. Search words were adapted to each database. Searches were limited to randomized trials. The full search strategy for each database is provided in Additional file 2. We searched for trials in two rounds: at the beginning of the review process and just before completion. The final search was performed 5 March 2014.

We also searched for relevant trials in citations from identified papers and former reviews. In addition, unpublished results from included trials were obtained from contact with authors. There was no language or publication date restriction.

### Eligibility criteria

Eligible studies included individually randomized trials, including quasi-randomized trials, and cluster-randomized trials.

#### Setting

Preparation for birth and parenthood are dependent on culture and contextual factors, such as the organization of the health care system. Trials taking place in developing countries have therefore been excluded and only trials conducted in the Western world - defined as OECD membership countries - are included [11].

#### Participants

We have included studies of pregnant women and/or their partners that have provided their informed consent to participation in the given trial or where descriptions in the papers indicate the participants’ consent to randomization.

#### Experimental and control conditions

The experimental conditions in the included trials must be delivered as an antenatal educational program offered by an educator to groups consisting of more than one individual/couple but including less than 20 individuals, related to delivery and/or preparation for parenthood. The control conditions in the included trials are either standard care, e.g., individual care only or other types of educational programs, e.g., antenatal education programs with a smaller intervention dose than the experimental condition. In cases where two programs were compared, the most intensive was considered the experimental intervention. Co-interventions were allowed only if the intervention was delivered equally in both the experimental and control arm.

#### Outcome measures

We included trials reporting quantitative outcome data. Outcome data from registers, self-report, or data reported by health professionals were accepted. In trials where outcomes were measured more than one time during follow-up, we have used the measurements shortly after the intervention ends and at the longest relevant follow-up to consider the intervention effect.

In trials where an outcome was measured by the same measurement tool at the same time point and reported both as a dichotomized result (RR) and as mean of scale, we have chosen to report the mean difference as the outcome.

The primary outcomes are as follows:

- Pain relief during labor.
- Obstetric interventions.
- Psychological and social adjustment to parenthood.
- Antenatal and postnatal depression and anxiety.

The secondary outcomes are as follows:

- Knowledge acquisition.
- Maternal sense of control/active decision-making during labor and birth.
- Partner involvement at birth.
- Breast feeding success.
- Infant care abilities.
- Social support.
- Relationship satisfaction.
- Divorce/separation.

### Study selection and data extraction

We conducted the selection of studies in two steps. First, two of three review authors (CSB, SFAX, and VK) independently performed the initial screening of all titles and abstracts to determine eligibility of all studies identified through the literature search. Next, two of three review authors (CSB, SGL, and VK) independently assessed the full papers identified as meeting inclusion criteria or where definite decision on exclusion could not be made from screening titles and abstracts. Any discrepancies between the assessors were resolved through discussion. A flow diagram of the selection process is shown in Additional file 3.

In some trials, the experimental and control condition received the exact same dose of antenatal education in small classes. These trials were excluded due to the difficulty of assessing the effect of antenatal education in small classes as an experimental condition as only the content varied between the experimental and the control condition.

Trials in which the experimental group received home visits, extra individual sessions, or presents for achieving the outcome in addition to the antenatal education classes were excluded as these co-interventions might have influenced the effect of the intervention beyond the effect of the classes. Extra written material to the experimental group was accepted. In trials where the intervention was ‘boosted’ by later individual consultations, we have used the measurement shortly before the individual consultation to consider the effect.

In cases where the content of the experimental or control condition was unclear or information incomplete, we contacted the first author by e-mail. We contacted 19 authors and received supplementary information from six of these.

Data from the included trials were extracted to summary tables containing information on the following: study design, inclusion and exclusion criteria, description of the experimental and control conditions, and outcomes of interest to the review.

### Risk of bias assessment

Two review authors (CSB and VK) independently assessed the included trials according to a predefined risk of bias scoring key [8] in order to determine the likely presence or absence of biases which might have affected the internal validity of the trials. Any discrepancies were resolved through discussion.

The scoring key includes the following characteristics:

- *Selection bias*: randomization sequence generation and allocation concealment.
- *Performance bias*: assessment of blinding of participants, educators, and outcome assessors. In trials where both subjective and objective outcomes are reported, we assessed blinding of outcome assessors separately for subjective and objective outcomes.
- *Incomplete outcome data*: assessment of systematic differences in withdrawal of study participants between the groups compared. In trials where both subjective and objective outcomes were reported, we assessed reporting bias separately for subjective and objective outcomes.
- *Selective outcome reporting bias*: assessment of systematic differences between reported and unreported findings. It was assessed whether a trial protocol exists and whether outcomes in the published trial had been reported in a pre-specified way.
- *Other sources of bias*: We assessed whether the trial was free of other sources of bias (e.g., baseline imbalance, recall bias).

First, each trial was evaluated according to each of the above-mentioned bias domains as either ‘low’ , ‘unclear’ , or ‘high risk of bias’. Secondly, the trials were rated by an overall risk of bias. All trials rated as ‘low risk of bias’ in all domains were scored ‘overall low risk of bias’. All other trials were scored ‘overall high risk of bias’. Due to the nature of the intervention, we expected a high level of bias for the domain ‘blinding of participants and educators’ as it is often not possible to blind participants and educators. If all trial bias domains were rated as ‘low risk of bias’ with the exception of ‘blinding of participants and educators’ , the trial was categorized as overall ‘moderate risk of bias’.

‘Risk of bias’ tables, ‘risk of bias summary’ , and ‘risk of bias graph’ for the included trials are shown in Additional file 4.

### Evidence synthesis

Structured summaries of the included trials are presented in ‘Characteristics of included trials’ in Additional file 5. Intervention effects from the included trials are calculated and presented as risk ratios (for dichotomous outcomes) or mean differences (for continuous outcomes) with 95% confidence intervals and two-sided *P* values for each outcome and reported in effect tables [see Additional file 6] and as forest plots [see Additional file 7]. A meta-analysis was planned beforehand [9]. However, due to diverse content of experimental as well as control conditions, this was not possible to perform.

### Protocol modifications

In addition to the pre-specified outcomes reported in the protocol [9], we have added relationship satisfaction and divorce/separation as secondary outcomes as these outcomes are also of great relevance as psycho-social dimensions of becoming parents.

We have reported mean differences as effect measures for continuous outcomes instead of standardized mean differences as defined by the protocol. In the process of conducting the systematic review, we found that meta-analyses could not be performed. Therefore, mean differences were preferred.

In the risk of bias assessment tool, we have included the score ‘overall moderate risk of bias’ for trials free of all other bias other than blinding of participants and educators and assessed the risk of bias separately for objective and self-reported outcomes.

## Results

### Description of included trials

We identified 5,708 records from the literature searches and an additional ten records were identified from reference lists and contact to author. A detailed flow diagram of the study selection process is shown in Additional file 3. In total, we included 17 trials in the review. We have provided full details of the included trials in the ‘Characteristics of included trials’ table [see Additional file 5]. A list of excluded trials with brief explanation of reasons is reported in Additional file 8.

Some trials were reported in more than one report. The 17 trials were reported in 21 papers and 1 oral presentation. Only papers fulfilling the requirements for analysis are included. The trial by Maimburg et al. was reported in two papers and one oral presentation [12-14], and the trial by Werner was reported in three papers [15-17]. For the remainder of the review, only the main report for each included trial is cited.

Results from the included trials were reported between 1988 and 2014 in 20 papers and 1 oral presentation (obtained from the author). Six trials were conducted in the United States [18-23], four trials were conducted in Australia [24-27], two were conducted in Denmark [12,16], one in the United Kingdom [28], one in Canada [29], one in Finland [30], one in Mexico [31], and one multicenter trial was conducted in Spain and France [32]. In total, the trials included 6,507 randomized women and 961 men, with a range from 74 to 1,193 participants per trial.

All trials tested the effect of antenatal education in small classes; however, the content and form of the experimental condition varied between the trials. The amount of education in the experimental condition varied from a single 1-h session [24] to 24 sessions each lasting 2.5 h [22]. Some trials focused on prevention of a specific condition among participants at specific risk, e.g., women at high risk of postnatal depression [21,28,31] or women with low socio-economic status [19,32]. Other interventions were targeted at a broader population group, e.g., all *primipara* at a specific birth site [12]. Also, control conditions differed between trials. In most of the trials, the control group was offered standard care which varied by content and amount, e.g., individual consultations with a midwife that also the experimental condition was offered [12,31]. In four trials, the control group was offered other interventions other than antenatal classes, e.g., one-to-one contact with a medical doctor [19].

Two trials were directed towards expecting fathers [23,26], and three trials specifically addressed the couple as a unit [18,22,32]. The remainders of the trials were directed towards the pregnant women, but in some of them, the expecting fathers were welcome to join one or all sessions.

For three of the pre-specified outcomes, maternal sense of control/active decision-making during labor and birth, partner involvement at birth, and infant care abilities, no data were reported. Data on pain relief during labor, obstetric interventions, knowledge acquisition, breast feeding, social support, relationship quality and divorce/separation, and psychological and social adjustment to parenthood were reported. Within the overarching category of psychological and social adjustment to parenthood, the following outcomes have been reported: antenatal and postnatal depression, anxiety, readiness for delivery and child care, self-efficacy and locus of control, co-parenting, and parent-child interaction.

### Risk of bias in included trials

We assessed the risk of bias in the 17 included trials. Full details on the risk of bias scoring can be found in the ‘risk of bias tables’ , ‘risk of bias summary’ , and ‘risk of bias graph’ [see Additional file 4]. All trials except for two [27,30] reported self-reported outcomes, and two trials additionally reported objective outcomes [12,16]. Blinding of participants was only possible in one trial [32].

All trials were scored overall ‘high risk of bias’ for the self-reported outcomes. For the objective outcomes, two trials were scored ‘overall moderate risk of bias’ [12,16]. These two trials were scored ‘overall high risk of bias’ for the self-reported outcomes since participants were not blinded. Also, the trial by Ickovics et al. was scored ‘overall high risk of bias’ for the same reason although this trial had ‘low risk of bias’ in all other domains but reported no objective outcomes [20].

### Effects of interventions

#### Depression prevention classes versus standard care

Three trials compared a depression-preventive program in small classes with standard care [21,28,31]. Brugha et al. examined the effect of a depression prevention antenatal program for women at risk of depression and found no significant effect on depression measured with several different measurement tools, self-efficacy, or locus of control [28]. A trial conducted by Lara et al. examined effects of a psycho-educational antenatal program among women at high risk of depression and reported no effect on depressive symptoms 6 weeks postnatally [31]. Also, Le et al. reported no effect of a psycho-educational antenatal program among women at high risk of depression - neither in pregnancy nor 6 weeks postnatally [21]. All three trials were scored ‘overall high risk of bias’.

#### Psycho-social prevention program versus brochure on child care

One trial assessed the effect of a psycho-social prevention program for couples, compared to a brochure on child care delivered to participants in the control condition [18] on depressive symptoms, co-parenting, anxiety, and parent-child interaction for both mothers and fathers 6 months postnatally. They reported that fathers, but not mothers, in the experimental group experienced significantly higher co-parental support (MD 0.29, 0.05 to 0.53), parenting-based closeness (MD 0.35, 0.04 to 0.66), and significantly lower father-child dysfunctional interaction (MD−0.26, −0.43 to −0.09) compared to fathers in the control condition [18]. This trial was scored ‘overall high risk of bias’.

#### Psycho-educational classes versus letter on fear of childbirth

One trial by Rouhe et al. compared the effect a group-based psycho-educational intervention directed towards women with severe fear of childbirth to written information in the form of a letter addressing fear of childbirth delivered to the participants in the control condition [30]. They found that the intervention significantly increased the likelihood of spontaneous vaginal delivery (RR 1.33, 1.11 to 1.61). They reported no effect on the use of epidural analgesia, overall caesarean section, elective and emergency caesarean section, vacuum extraction, and induction of labor [30]. This trial was scored ‘overall high risk of bias’.

#### Program using a psycho-somatic approach versus standard antenatal education program

Ortiz Collado et al. examined the effect of an antenatal psychosomatic program designed to decrease depression among women at high risk of postnatal depression compared to standard care [32]. They reported no significant effect on depression, social support, or relationship satisfaction among women. They also assessed relationship satisfaction among men and reported no significant effect [32]. This trial was scored ‘overall high risk of bias’.

#### Couple-focused classes versus standard care

One trial by Schulz et al. assessed the effect of a couple-focused intervention compared to standard care on marital satisfaction among both mothers and fathers 6 months and 5.5 years postnatally as well as divorce/separation 5.5 years postnatally. They reported no significant intervention effects on any of these outcomes [22]. This trial was scored ‘overall high risk of bias’.

#### Self-hypnosis classes versus standard care

Werner et al. compared a self-hypnosis intervention with standard care and reported no effect on the outcomes: use of epidural analgesia as pain relief during labor, spontaneous delivery, overall caesarean section, elective caesarean section, vacuum extraction, oxytocin augmentation, induction of labor, and any breast feeding 4 months postnatally [16]. However, they reported a statistically significant increased risk of emergency caesarean section (RR 1.52, 1.02 to 2.27) in the experimental group [16]. For the outcomes related to delivery, this trial was scored ‘overall moderate risk of bias’ , while the score was ‘overall high risk of bias’ for breast feeding which was self-reported.

#### General antenatal education classes versus standard care

One trial by Maimburg et al. assessed the effect of general group-based antenatal training among primiparous compared to standard care on a range of both pharmacological and non-pharmacological pain relief outcomes, obstetric interventions, postnatal depression, breast feeding, breast feeding knowledge, and breast feeding self-efficacy [12]. They reported a protective effect on the use of epidural analgesia (RR 0.84, 0.73 to 0.98) but no significant effect on any other kind of pain relief or obstetric interventions, e.g., caesarean section and vacuum extraction. Also, no significant effects were reported on breast feeding at 5 weeks or 6 months postnatally and breast feeding self-efficacy or postnatal depression 6 weeks after birth. They reported a higher proportion with sufficient knowledge about breast feeding 6 weeks postnatally among women attending the general antenatal training program in small classes (RR 1.08, 1.01 to 1.15) [12]. For the outcomes related to delivery, this trial was scored ‘overall moderate risk of bias’ , while the score was ‘overall high risk of bias’ for breast feeding, breast feeding self-efficacy, knowledge, and postnatal depression which were self-reported.

#### Group prenatal care (20 h) versus individual prenatal care (2 h)

A trial by Ickovics et al. examined the effect of a general antenatal education program in small classes compared to individual prenatal care (total amount of time: 2 h) [20]. They reported significantly higher scores on prenatal and infant care knowledge (MD 2.60, 1.68 to 3.52) and readiness for labor and delivery (MD 7.60, 3.34 to 11.86) at 35-weeks gestation among women in the experimental condition. They found no effect on readiness for infant care or prenatal distress at 35-weeks gestation [20]. This trial was scored ‘overall high risk of bias’ due to the self-report of outcomes.

#### Paternal education class versus standard care

Two trials examined the effect of paternal education compared to standard care [23,26]. Westney et al. conducted an intervention targeted at prospective adolescent fathers. This intervention had a significantly positive effect on paternal knowledge acquisition in relation to pregnancy, delivery, infant care, and support towards the mother (MD 9.55, 1.25 to 17.85) [23]. Maycock et al. conducted a breast feeding intervention targeted at expecting fathers. They reported a significant intervention effect on any breast feeding 6 weeks postnatally (RR 1.09, 1.00 to 1.18). There was no effect on exclusive breast feeding 6 weeks postnatally [26]. Both of these trials were scored ‘overall high risk of bias’.

#### Extra breast feeding sessions versus standard care

In three trials, the authors examined the effect of giving extra breast feeding sessions in small classes [24,25,29]. Duffy et al. examined the effect of an antenatal group-teaching session aimed at increasing breast feeding prevalence but also reported obstetric interventions. They reported no effect on vaginal delivery, caesarean section, vacuum extraction, or forceps. They also assessed the effect on breast feeding and reported a positive effect on exclusive breast feeding 6 weeks postnatally (RR 3.20, 1.88 to 5.46) [24]. Noel-Weiss et al. examined effects of a breast feeding education workshop and reported no significant effect on breast feeding 8 weeks postnatally. However, they found a significantly higher breast feeding self-efficacy among participants in the experimental condition 4 weeks postnatally (MD 4.60, 0.72 to 8.48) but not 8 weeks postnatally [29]. Forster et al. conducted a trial comparing two breast feeding education classes with usual care. They reported no significant effect in initiation of breast feeding or breast feeding 6 months postnatally [25]. All three trials were scored ‘overall high risk of bias’.

#### Breast feeding classes versus one-to-one contact on breast feeding

Kistin et al. assessed the effect of a breast feeding class with group discussion compared to 15- to 30-min one-to-one contact with a medical doctor on breast feeding topics and reported no effect on initiation of breast feeding or of any breast feeding 12 weeks postnatally [19]. This trial was scored ‘overall high risk of bias’.

#### Breast feeding classes versus breast feeding and childbirth pamphlets

One trial assessed the effect of a breast feeding education program compared to breast feeding and childbirth pamphlets [27]. Rossiter reported a significantly higher rate of breast feeding initiation (RR 1.86, 1.35 to 2.55) among participant in the experimental condition but found no effect on breast feeding 6 months postnatally [27]. This trial was scored ‘overall high risk of bias’.

## Discussion

In this systematic review, we assessed the literature on the effect of antenatal education in small classes on obstetric and psycho-social outcomes. Across trials, the experimental and control conditions varied greatly both in their format and content, and therefore, we analyzed effect of interventions in effectively 12 different comparison groups across the 17 randomized controlled trials included. Many interventions were addressed by only one trial and the remaining in only a few trials. Due to the heterogeneity of the experimental and control conditions and outcomes, it was not appropriate to conduct meta-analysis. Most of the included trials reported on more than one outcome, and only a small number of outcomes showed statistically significant differences between the experimental and control condition. Furthermore, we found great inconsistency of results across studies, and there was no clear pattern of effect. For example, one trial assessing the effect of extra breast feeding sessions reported a positive effect on breast feeding duration [24], whereas two trials did not find an effect [25,29]. In summary, it is not possible to draw definitive conclusions on the effect of small group antenatal education on obstetric and psycho-social outcomes based on this systematic review.

### Quality of the evidence

We included 17 trials. All of these were assessed as ‘overall high risk of bias’ for the self-reported outcomes. For the objective outcomes, two trials were scored ‘overall moderate risk of bias’ [12,16]. The internal validity of the results of this review is therefore limited. Also, generally sample sizes were small - 12 of the 17 trials were conducted with fewer than 400 individuals randomized. There was a tendency that the larger and more recent trials had fewer methodological limitations and were reported in more detail than the earlier trials with smaller sample sizes. There is a need for trial authors to report trials according to the CONSORT principles [33].

### Strengths and limitations

We used the Cochrane Handbook for Systematic Reviews of Interventions [8] as a guide for conducting this systematic review. We registered the review within the International Prospective Register of Systematic Reviews (PROSPERO) and published our methods as a protocol before conducting the review [9]. We conducted a thorough literature search performed by an information specialist and had no restrictions regarding language and publication date. Two review authors independently extracted data and scored risk of bias according to a detailed bias assessment tool.

The trials included in this review are very diverse regarding experimental conditions, control conditions, and populations studied and are therefore difficult to compare. The strength of the conclusions is limited by sparse and lower quality of evidence.

In 2007, a systematic review by Gagnon and Sandall was conducted [3] evaluating the effect of both individual and group antenatal education for childbirth or parenthood. They concluded that high-quality evidence was lacking and that the effects of antenatal education are largely unknown. In this review, we specifically focused on antenatal education in small classes conducted in a Western setting and assessed the literature up to 2014. Also, in the present review, we found limited evidence from which to draw conclusions regarding the effect of antenatal education in small classes. We chose to focus primarily on evaluating evidence about the form of antenatal education, i.e., education in small classes and not the content as such. We excluded trials evaluating two programs with the same dose of antenatal education in small classes. To look into the effect of content, it would be relevant to conduct a systematic review evaluating this aspect.

### Implications for research

There is a need to conduct high-quality, randomized trials with adequate sample sizes and transparent reporting of relevant outcome measures to evaluate the effect of antenatal education in small classes. Results from a large ongoing randomized trial will soon be available [34].Given the uncertainty in effects and costs of small group antenatal education, we would recommend that future trials should first focus on a comparison to standard care rather than comparing the relative effects of different educational programs. Future trials should also initially assess the feasibility of interventions in order that they develop and evaluate educational programs that are likely to be implementable in an everyday clinical practice setting, if proven effective. Finally, there is the issue of the trial population and whether to apply the educational intervention to the broad population or to limit it to high-risk populations, such as those with depression.

### Implications for practice

No clear recommendations for practice can be made based on the results of this review. The trials included all varied greatly in extent, method, and content, and a meta-analysis was not possible to perform. This makes it difficult to compare results across trials.

## Conclusions

Insufficient evidence exists as to whether antenatal education in small classes has any effect on obstetric or psycho-social outcomes. Given that the evidence base is inconclusive, emerging evidence from future well-conducted and well-reported trials may help to make conclusions about the effectiveness of antenatal education in small classes. We recommend updating this review regularly with emerging evidence.

## Additional files

Additional file 1:
**PRISMA 2009 checklist.** The file contains a filled in PRISMA checklist for the systematic review. Additional file 2:
**Search strategy.** The file contains the search strategy used in the databases Medline, EMBASE, CENTRAL, CINAHL, Web of Science, and PsycINFO. Additional file 3:
**PRISMA 2009 Flow Diagram.** The file contains a flow diagram of the trial selection process. Additional file 4:
**Risk of bias tables.** The file contains the assessment of risk of bias for each included trial, a risk of bias summary, a risk of bias graph. Additional file 5:
**Characteristics of included trials.** The file contains characteristics of design, participants, content of experimental and control conditions, and outcomes for each included trial. Additional file 6:
**Effect tables.** The file contains tables of measures of intervention effects (RR and MD) with 95% confidence intervals and two-sided *P* values for each outcome in the included trials. Additional file 7:
**Forest plots.** The file contains forest plots of intervention effects (RR and MD) for each outcome in the included trials. Additional file 8:
**Characteristics of excluded trials.** The file contains a list of excluded trials with brief explanations of reasons for exclusion.
