# Supplementary material for: The effect of antenatal education in small classes on obstetric and psycho-social outcomes - a systematic review
Source: Syst Rev. 2015 Feb 28;4:20. doi: 10.1186/s13643-015-0010-x (PMC4355374; doi:10.1186/s13643-015-0010-x)
Supplement: Additional file 2: — Search strategy. The file contains the search strategy used in the databases Medline, EMBASE, CENTRAL, CINAHL, Web of Science, and PsycINFO. [file 13643_2015_10_MOESM2_ESM.docx]

**Search strategy**

**Web of Science**

All indexes, all years. Filters: none.

1: TS=(antenatal OR prenatal OR pregnancy OR birth OR childbirth OR (labor OR labour) OR obstetric OR (delivery OR deliveries))

2: TS=(education OR "parent education*" OR preparation OR "parent preparation" OR "early intervention")

3: TS=("randomi* control* trial*" OR "randomi* trial*" OR "randomi* clinical trial*")

4: 1 AND 2 AND 3.

**Medline**

Filters: Refined by *randomized controlled trial*, *humans*.

1: TS=(antenatal OR prenatal OR pregnancy OR birth OR childbirth OR (labor OR labour) OR obstetric OR (delivery OR deliveries)) 2: TS=(education OR "parent education*" OR preparation OR "parent preparation" OR "early intervention") 3: 1 AND 2

**Cinahl**

Filters: Refined by *randomized controlled trial*.

1: SU= antenatal OR prenatal OR pregnancy OR birth OR childbirth OR (labor OR labour) OR obstetric OR (delivery OR deliveries))

2: SU=education OR "parent education*" OR preparation OR "parent preparation" OR "early intervention"

3: 1 AND 2

Additional Cinahl search with no filters:

4: SU=antenatal OR prenatal OR pregnancy OR birth OR childbirth OR (labor OR labour) OR obstetric OR (delivery OR deliveries)

5: SU=education OR "parent education*" OR preparation OR "parent preparation" OR "early intervention")

6: SU="randomi* control* trial*" OR "randomi* trial*" OR "randomi* clinical trial*"

7: 4 AND 5 AND 6

8: 3 AND 7

**Cochrane**

Filter: Title, abstract, keyword, refined by *trials*.

1: antenatal OR prenatal OR pregnancy OR birth OR childbirth OR (labor OR labour) OR obstetric OR (delivery OR deliveries))

2: (education OR "parent education*" OR preparation OR "parent preparation" OR "early intervention")

3: ("randomi* control* trial*" OR "randomi* trial*" OR "randomi* clinical trial*")

4: 1 AND 2 AND 3

**Embase / Psycinfo**

Embase and Psycinfo were searched together in the same database. No filters.

In total, four combined searches were made; in abstracts (AB), in keywords (key), in subject headings (SH), and in titles (TI).

1: AB: antenatal OR prenatal OR pregnancy OR birth OR childbirth OR (labor OR labour) OR obstetric OR (delivery OR deliveries)

2: AB: education OR "parent education*" OR preparation OR "parent preparation" OR "early intervention"

3 ALL FIELDS: "randomi* control* trial*" OR "randomi* trial*" OR "randomi* clinical trial*"

4: 1 AND 2 AND 3

5: KEY: antenatal OR prenatal OR pregnancy OR birth OR childbirth OR (labor OR labour) OR obstetric OR (delivery OR deliveries)

6: KEY: education OR "parent education*" OR preparation OR "parent preparation" OR "early intervention"

7: ALL FIELDS: "randomi* control* trial*" OR "randomi* trial*" OR "randomi* clinical trial*"

8: 5 AND 6 AND 7

9: SH: antenatal OR prenatal OR pregnancy OR birth OR childbirth OR (labor OR labour) OR obstetric OR (delivery OR deliveries)

10: SH: education OR "parent education*" OR preparation OR "parent preparation" OR "early intervention"

11: ALL FIELDS: "randomi* control* trial*" OR "randomi* trial*" OR "randomi* clinical trial*"

12: 9 AND 10 AND 11

13: TI: antenatal OR prenatal OR pregnancy OR birth OR childbirth OR (labor OR labour) OR obstetric OR (delivery OR deliveries)

14: TI: education OR "parent education*" OR preparation OR "parent preparation" OR "early intervention"

15: ALL FIELDS: "randomi* control* trial*" OR "randomi* trial*" OR "randomi* clinical trial*"

16: 13 AND 14 AND 15

17: 4 AND 8 AND 12 AND 16
