# Supplementary material for: The effect of antenatal education in small classes on obstetric and psycho-social outcomes - a systematic review
Source: Syst Rev. 2015 Feb 28;4:20. doi: 10.1186/s13643-015-0010-x (PMC4355374; doi:10.1186/s13643-015-0010-x)
Supplement: Additional file 3: — PRISMA 2009 Flow Diagram. The file contains a flow diagram of the trial selection process. [file 13643_2015_10_MOESM3_ESM.doc]

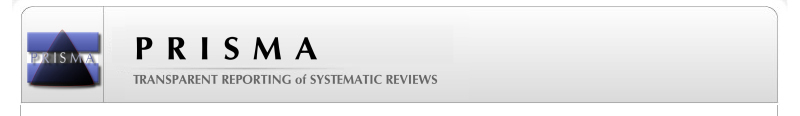
**PRISMA 2009 Flow Diagram**

**Screening**

**Included**

**Eligibility**

**Identification**

Records identified through database searching
(n = 8,903)

Additional records identified through other sources
(n = 10)

Records after duplicates removed
(n = 5,718)

Records screened
(n = 5,718)

Records excluded
(n = 5,631)

Full-text articles (+ presentations) assessed for eligibility
(n = 87)

Full-text articles excluded, with reasons (n=66)

Non-western country: 16

Non-randomized trial: 2

Outcome outside the scope of review: 11

Experimental condition outside the scope of review: 4

Individual-based interventions: 7

Same dose in experimental and control group: 9

Co-interventions: 9

Not in a form suitable for analysis (authors contacted for further information): 8

Trials included in qualitative synthesis
(n = 17)

Reported in 21 records
