# Supplementary material for: The effect of antenatal education in small classes on obstetric and psycho-social outcomes - a systematic review
Source: Syst Rev. 2015 Feb 28;4:20. doi: 10.1186/s13643-015-0010-x (PMC4355374; doi:10.1186/s13643-015-0010-x)

**Risk of bias**

**Brugha 2000**

**Overall risk of bias: high**

| **Risk of bias** | **Description** | **Judgement** |
| --- | --- | --- |
| **Sequence generation** | Computerized stratified randomization, using minimization on three prognostic factors: level of social support, screen GHQ-D and ethnic group, was used by the research interviewer to allocate half the consenting women to intervention and half to control | Low |
| **Allocation concealment** | No description. | Unclear |
| **Blinding of participants and educators** | Not possible to blind participants. The educators were not blinded but “…they were not involved in any way in intervention allocation or in the research assessments”. | High |
| **Blinding of outcome assessor (self-reported outcomes)** | Not possible to blind outcome assessors (self-reported outcomes).  Outcome assessors (interviewers) were blinded towards intervention group, and the women were asked not to reveal their status. “The allocation code was not broken until completion of the fieldwork and primary analyses.”  “Analysis of the interviewer's records of which group she thought each respondent had been allocated to showed no  difference from chance.” | High |
| **Incomplete outcome data**  **(self-reported outcomes)** | Nonresponse rate was 9 % in both the experimental and control condition. Sensitivity analyses testing the influence of these missing outcome data did not alter the results. | Low |
| **Selective reporting bias** | No study protocol found. All stated primary and secondary outcome measures stated in the paper are reported. | Low |
| **Other sources of bias** |  | Low |

**Duffy 1997**

**Overall risk of bias: high**

| **Risk of bias** | **Description** | **Judgement** |
| --- | --- | --- |
| **Sequence generation** | Randomized in blocks of 12 – no further description of sequence generation. | Unclear |
| **Allocation concealment** | Group allocation was blinded to the researcher. “Randomization was achieved using a sealed envelope  technique….”. | Low |
| **Blinding of participants and educators** | Not possible to blind participants or educators. | High |
| **Blinding of outcome assessor (self-reported outcomes)** | Not possible to blind outcome assessors (self-reported outcomes). | High |
| **Incomplete outcome data**  **(self-reported outcomes)** | Two women in the experimental group were excluded from data analysis due to revelation of treatment condition.  3 women in the control condition excluded. No information on differences in characteristics. Full response rate on the remaining – 35 participants in each condition. | Low |
| **Selective reporting bias** | No study protocol found, but all listed primary outcomes in the paper are reported. | Unclear |
| **Other sources of bias** |  | Unclear |

**Feinberg 2008**

**Overall risk of bias: high**

| **Risk of bias** | **Description** | **Judgement** |
| --- | --- | --- |
| **Sequence generation** | No description of procedure | Unclear |
| **Allocation concealment** | No description | Unclear |
| **Blinding of participants and educators** | Not possible to blind participants or educators. | High |
| **Blinding of outcome assessor (self-reported outcomes)** | Not possible to blind outcome assessors (self-reported outcomes). | High |
| **Incomplete outcome data**  **(self-reported outcomes)** | No differential drop-out rates at 6 months follow-up; 11 % in the experimental and 9 % in the control condition. There was no evidence of differential attrition by condition. | Low |
| **Selective reporting bias** | Reporting of results in 3 papers. No indication of relevant outcomes not reported. | Low |
| **Other sources of bias** |  | Unclear |

**Forster 2004**

**Overall risk of bias: high**

| **Risk of bias** | **Description** | **Judgement** |
| --- | --- | --- |
| **Sequence generation** | “A computerized system of biased urn randomization” | Low |
| **llocation concealment** | Randomization “was accessed by telephone by the research midwife to ascertain women’s group allocation”. | Low |
| **Blinding of participants and educators** | Not possible to blind participants or educators. | High |
| **Blinding of outcome assessor (self-reported outcomes)** | Not possible to blind outcome assessors (self-reported outcomes). | High |
| **Incomplete outcome data**  **(self-reported outcomes)** | Non-response rate for experimental group: 5 % at 2-4 days after birth and 11 % at 6 months. Control group: 5 % at 2-4 days after birth and 9 % at 6 months. No reporting on differences in characteristics of non-responders between groups. | Unclear |
| **Selective reporting bias** | According to study protocol all listed primary outcomes are reported. | Low |
| **Other sources of bias** | 35 women were unable to be interviewed at first follow-up. They answered questions later – may cause recall bias. No information on differences in traceability of respondents between conditions given. | Unclear |

**Ickovicks 2007**

**Overall risk of bias: high**

| **Risk of bias** | **Description** | **Judgement** |
| --- | --- | --- |
| **Sequence generation** | “A computer-generated randomization sequence, password  protected to recruitment staff and participants, was used to assign participants”. | Low |
| **Allocation concealment** | “Allocation was concealed from participant and research staff until eligibility screening was completed and study condition was assigned. These tasks were completed by trained research team members who were independent of prenatal care”. | Low |
| **Blinding of participants and educators** | Not possible to blind patients and educators. | High |
| **Blinding of outcome assessor (self-reported outcomes)** | Not possible to blind outcome assessors (self-reported outcomes). | High |
| **Incomplete outcome data**  **(self-reported outcomes)** | No differential drop-out between experimental (10 %) and control group (11.5 %) in the measurements in week 35. No reported drop-out number for breast-feeding initiation. | Low |
| **Selective reporting bias** | No study protocol found, but all listed primary outcomes in the paper are reported. | Low |
| **Other sources of bias** | There were differences in some of the baseline characteristics. Authors made analyses adjusted for these variables. This did not change the overall significance level. | Low |

**Kistin 1990**

**Overall risk of bias: high**

| **Risk of bias** | **Description** | **Judgement** |
| --- | --- | --- |
| **Sequence generation** | “Women… were randomly, using a random numbers table,…” | Low |
| **Allocation concealment** | No description. | Unclear |
| **Blinding of participants and educators** | Not possible to blind patients and educators. | High |
| **Blinding of outcome assessor (self-reported outcomes)** | Not possible to blind outcome assessors (self-reported outcomes). | High |
| **Incomplete outcome data**  **(self-reported outcomes)** | From the information given it is not possible to calculate drop-out rates for each of the two groups included in the review. Overall, of the 159 women who agreed to participate, 29 dropped out (18 %). Drop-outs differed in age and breast feeding plans (not significant). | High |
| **Selective reporting bias** | No study protocol found. No outcomes other than the reported are listed. | Unclear |
| **Other sources of bias** | Large differences in some of the baseline characteristics related to the outcome. | High |

**Lara 2010**

**Overall risk of bias: high**

| **Risk of bias** | **Description** | **Judgement** |
| --- | --- | --- |
| **Sequence generation** | Randomization was performed using a blocked randomization procedure. Blocks were sequentially opened every time a group started. To ensure conditions were balanced, an envelope contained equal number of folded papers for each one for the first two groups. | Low |
| **Allocation concealment** | No description | High |
| **Blinding of participants and educators** | Not possible to blind participants or educators. | High |
| **Blinding of outcome assessor (self-reported outcomes)** | Not possible to blind outcome assessors (self-reported outcomes). | High |
| **Incomplete outcome data**  **(self-reported outcomes)** | Non-response rate were much higher in the experimental group (72 %) 6 weeks after birth than in the control group (39 %). No reporting on differences in characteristics of non-responders between groups. | High |
| **Selective reporting bias** | No study protocol found. No other outcomes are listed in the paper. | Unclear |
| **Other sources of bias** | Women in the intervention group had higher rates of depressive symptoms and anxiety at baseline. | High |

**Le 2011**

**Overall risk of bias: high**

| **Risk of bias** | **Description** | **Judgement** |
| --- | --- | --- |
| **Sequence generation** | No description | Unclear |
| **Allocation concealment** | “… a sealed envelope with her group membership was assigned by the first author; neither participant nor interviewer knew the result of the random assignment until this envelope was opened.” | Low |
| **Blinding of participants and educators** | Not possible to blind participants or educators. | High |
| **Blinding of outcome assessor (self-reported outcomes)** | Not possible to blind outcome assessors (self-reported outcomes). Interviewers at each outcome assessment time point were not blind to group assignment. | High |
| **Incomplete outcome data**  **(self-reported outcomes)** | Nonresponse rate at the post intervention assessment was 16 % in the experimental group and 12 % in the control condition. At 6 weeks postpartum, the nonresponse rate was higher in the experimental group (21 %) than in the control group (13 %). No data on differences in characteristics are presented. | High |
| **Selective reporting bias** | No study protocol found. No other outcomes are listed in the paper. | Unclear |
| **Other sources of bias** |  | Unclear |

**Maimburg 2010**

**Overall risk of bias:**

*Objective outcomes: moderate*

*Self-reported outcomes: high*

| **Risk of bias** | **Description** | **Judgement** |
| --- | --- | --- |
| **Sequence generation** | “The randomization program used an algorithm generated by a data manager”. | Low |
| **Allocation concealment** | “Randomization was assigned by a staff midwife using a  computer-assisted voice response system” | Low |
| **Blinding of participants and educators** | Not possible to blind participants and educators. | High |
| **Blinding of outcome assessor** | | |
| *Objective outcomes* | Outcome assessors (midwives in the maternity ward) were blinded towards intervention group. | Low |
| *Self-reported outcomes* | Not possible to blind outcome assessors (self-reported outcomes). | High |
| **Incomplete outcome data** | | |
| *Objective outcomes* | In both the experimental and control group non-report on obstetric outcomes were 3 %. | Low |
| *Self-reported outcomes* | In the measure of breast-feeding at 6 weeks the non-response rate for both groups were around 10 %. No data on differences in characteristics are presented. | Unclear |
| **Selective reporting bias** | All main outcomes reported in study protocol are reported in the main paper of the study. The secondary outcomes are reported in other papers or oral presentations. | Low |
| **Other sources of bias** | Sample size calculations based on the primary outcome. | Low |

**Maycock 2013**

**Overall risk of bias: high**

| **Risk of bias** | **Description** | **Judgement** |
| --- | --- | --- |
| **Sequence generation** | Randomization with a random number generator. | Low |
| **Allocation concealment** | Participants were randomized, with no blinding | High |
| **Blinding of participants and educators** | Not possible to blind participants or educators. | High |
| **Blinding of outcome assessor (self-reported outcomes)** | Not possible to blind outcome assessors (self-reported outcomes). | High |
| **Incomplete outcome data**  **(self-reported outcomes)** | Not possible to calculate non-response rates for each group separately. | Unclear |
| **Selective reporting bias** | No study protocol found. The primary outcome is reported. | Unclear |
| **Other sources of bias** | Sample size calculations based on the primary outcome. | Low |

**Noel-Weiss 2006**

**Overall risk of bias: high**

| **Risk of bias** | **Description** | **Judgement** |
| --- | --- | --- |
| **Sequence generation** | Sequentially numbered envelopes | Unclear |
| **Allocation concealment** | “Participants returned the registration package in a sealed manila envelope, and randomization was completed by matching the manila envelope with a sealed, sequentially numbered, opaque envelope containing a slip of paper stating either Control or Workshop” | Low |
| **Blinding of participants and educators** | Not possible to blind participants or educators. | High |
| **Blinding of outcome assessor**  **(self-reported outcomes)** | Not possible to blind outcome assessors (self-reported outcomes). | High |
| **Incomplete outcome data**  **(self-reported outcomes)** | Not possible to calculate the non-response rate for each group separately. | Unclear |
| **Selective reporting bias** | No study protocol found, but all listed primary outcomes in the paper are reported. | Unclear |
| **Other sources of bias** |  | Low |

**Ortiz Collado 2014**

**Overall risk of bias: high**

| **Risk of bias** | **Description** | **Judgement** |
| --- | --- | --- |
| **Sequence generation** | ”A statistician produced a computer generated random distribution of women with antenatal risk of PPD in both groups, EG and CG”. | Low |
| **Allocation concealment** | “The allocation to the study groups was blinded; all interviews were sent to an outside statistician…. The statistician telephoned the researcher to notify the assignment of eligible women to control groups or experimental groups”. | Low |
| **Blinding of participants and educators** | “Participants knew they were in a study group but did not know the distinction between control and experimental intervention. The nurse midwives who ran the control group also had no prior knowledge. Only nurse midwives who animated the experimental group knew about the distinction but never had access to the questionnaires and never knew the evaluated variables.” | Low |
| **Blinding of outcome assessor (self-reported outcomes)** | Outcome assessors (self-reported outcomes) were blinded. | Low |
| **Incomplete outcome data**  **(self-reported outcomes)** | Non-response rate for the questionnaire were higher in the control group (36 %) than in the experimental group (25 %). No data on differences in characteristics are presented. | High |
| **Selective reporting bias** | No study protocol found. Both the stated primary and secondary outcome stated in the paper are reported. | Unclear |
| **Other sources of bias** |  | Low |

**Rossiter 1994**

**Overall risk of bias: high**

| **Risk of bias** | **Description** | **Judgement** |
| --- | --- | --- |
| **Sequence generation** | No description of procedure. Large differences in number of participants assigned to the two conditions. | High |
| **Allocation concealment** | No description. | Unclear |
| **Blinding of participants and educators** | Not possible to blind participants or educators. | High |
| **Blinding of outcome assessor (objective outcomes)** | No description | Unclear |
| **Incomplete outcome data (objective outcomes)** | Non-response rates: post-test: 6 %, 6 months following birth: 10 %. No reporting on non-response rates for experimental and control groups separately. | Unclear |
| **Selective reporting bias** | No study protocol found, but all listed primary outcomes in the paper are reported. | Unclear |
| **Other sources of bias** | Large differences in baseline characteristics related to the outcome which may have biased the results. | High |

**Rouhe 2012**

**Overall risk of bias: high**

| **Risk of bias** | **Description** | **Judgement** |
| --- | --- | --- |
| **Sequence generation** | No description. | Unclear |
| **Allocation concealment** | “.. were randomised … to the intervention or control group in the proportion of 1:2 in balanced blocks of 18 by sealed opaque envelopes.” | Low |
| **Blinding of participants and educators** | Not possible to blind participants or educators. | High |
| **Blinding of outcome assessor (objective outcomes)** | The outcomes were assessed from medical records. | Low |
| **Incomplete outcome data (objective outcomes)** | Full response rate. | Low |
| **Selective reporting bias** | No study protocol found. The listed primary outcome is reported. | Unclear |
| **Other sources of bias** | Discrepancies between numbers of randomized women in the two papers reporting from the study. | High |

**Schulz 2006**

**Overall risk of bias: high**

| **Risk of bias** | **Description** | **Judgement** |
| --- | --- | --- |
| **Sequence generation** | “Expectant couples were randomized to condition using a random number table..” | Low |
| **Allocation concealment** | No description. | Unclear |
| **Blinding of participants and educators** | Not possible to blind participants or educators. | High |
| **Blinding of outcome assessor (self-reported outcomes)** | Not possible to blind outcome assessors (self-reported outcomes). | High |
| **Incomplete outcome data**  **(self-reported outcomes)** | 94 % returned the 6 month follow-up questionnaire – 96 % in the experimental group and 92 % in the control group. At the 66 months follow-up 46 % in the experimental and 66 % in the control group returned questionnaire. No information on differences in characteristics among responders and non-responders are given. The drop-out rates at 66 months are substantial and differ between groups. | High |
| **Selective reporting bias** | No specification of other collected outcomes in the trial. | Unclear |
| **Other sources of bias** |  | Unclear |

**Werner 2013**

**Overall risk of bias:**

*Objective outcomes: moderate*

*Self-reported outcomes: high*

| **Risk of bias** | **Description** | **Judgement** |
| --- | --- | --- |
| **Sequence generation** | Computer-generated system. | Low |
| **Allocation concealment** | “The participants were randomly allocated… using a  computer-generated interactive voice response telephone  randomization system”. | Low |
| **Blinding of participants and educators** | Not possible to blind participants or educators. | High |
| **Blinding of outcome assessor** | | |
| *Objective outcomes* | Outcome assessor for the birth related outcomes (midwives assisting the birth) were blinded to the participant’s allocated treatment. Outcomes were extracted from an ongoing data collection from all births at the hospital or from medical records. | Low |
| *Self-reported outcomes* | Not possible to blind outcome assessors (self-reported outcomes). | High |
| **Incomplete outcome data** | | |
| *Objective outcomes* | Full response rate | Low |
| *Self-reported outcomes* | Response rate for the 6 week questionnaire were high (97 % and 98.4 % in control group and experimental group respectively). At 6 months after birth the corresponding rates were 96.1 % and 96.8 %. No data on differences in characteristics are presented, but very low drop-out rate in both conditions. | Low |
| **Selective reporting bias** | Study protocol available. The stated primary outcome is reported, and only a few secondary outcomes are not yet reported. | Low |
| **Other sources of bias** |  | Low |

**Westney 1988**

**Overall risk of bias: high**

| **Risk of bias** | **Description** | **Judgement** |
| --- | --- | --- |
| **Sequence generation** | No description of randomization procedure. | Unclear |
| **Allocation concealment** | No description. | Unclear |
| **Blinding of participants and personnel** | Not possible to blind participants and educators | High |
| **Blinding of outcome assessor (self-reported outcomes)** | Not possible to blind outcome assessors (self-reported outcomes). | High |
| **Incomplete outcome data**  **(self-reported outcomes)** | No drop-out from baseline to follow-up. | Low |
| **Selective reporting bias** | No study protocol found. No outcomes other than the reported are listed. | Unclear |
| **Other sources of bias** |  | Unclear |

**Figure 4.a. Risk of bias summary: review authors' judgments about each risk of bias item for each included study**

**
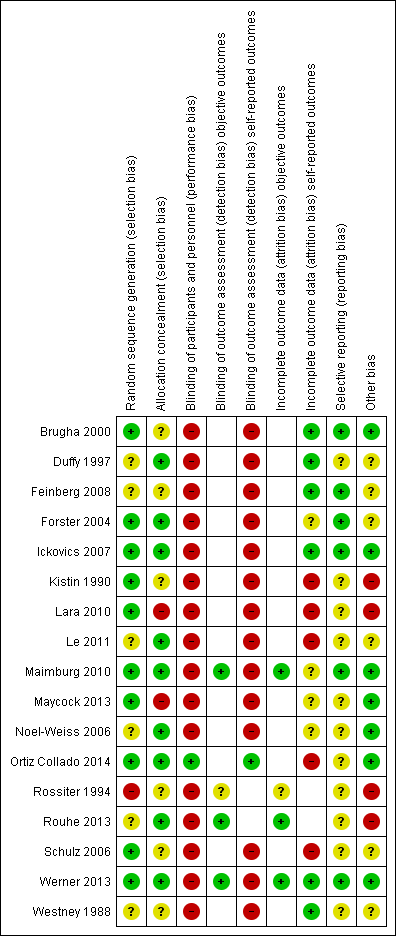
**

**Figure 4.b. Risk of bias graph: review authors' judgments about each risk of bias item presented as percentages across all included studies**


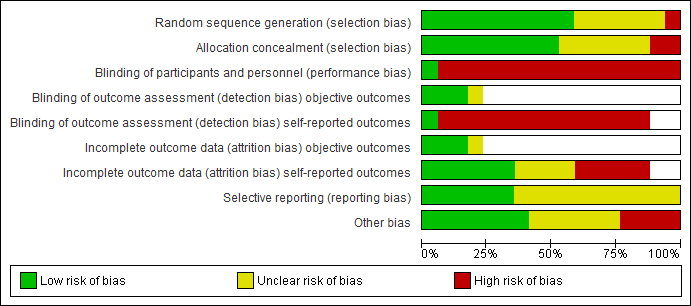

Supplement: Additional file 4: — Risk of bias tables. The file contains the assessment of risk of bias for each included trial, a risk of bias summary, a risk of bias graph. [file 13643_2015_10_MOESM4_ESM.docx]
