# Supplementary material for: The effect of antenatal education in small classes on obstetric and psycho-social outcomes - a systematic review
Source: Syst Rev. 2015 Feb 28;4:20. doi: 10.1186/s13643-015-0010-x (PMC4355374; doi:10.1186/s13643-015-0010-x)
Supplement: Additional file 5: — Characteristics of included trials. The file contains characteristics of design, participants, content of experimental and control conditions, and outcomes for each included trial. [file 13643_2015_10_MOESM5_ESM.docx]

**Characteristics of included trials**

|  | **Design** | **Participants** | **Experimental and control conditions** | **Outcomes of interest to the review** |
| --- | --- | --- | --- | --- |
| Brugha 2000 | Individually randomized trial conducted in Leicester, UK. | 209 women in risk of postnatal depression.  Experimental (n=103), control (n=106).  **Inclusion criteria:** pregnant woman screened at risk of postnatal depression, at least 16 years of age, primipara, planning to continue pregnancy to full-term, residing within reasonable travelling distance of the hospital, capable of understanding and completing screening questionnaires in English and of giving written, informed consent. | **Experimental condition**:   - A depression-preventive program consisting of 6 structured 2-hour weekly antenatal classes and a postnatal reunion class. - Starting in week 28 of gestation. - Group size was 8-16 women. - General education was given rather than formal lectures – both discussion exercises and role plays were used. The group was expected to provide emotional support. The woman's partner or significant other was encouraged to attend one session. - Instructors were nurses and occupational therapists, with extensive experience in hospital and community general psychiatry.   **Control condition:** Standard care consisting of 10 individual consultations with a community midwife. No specific focus on depression prevention. | All outcomes were assessed three months postnatal with self-completion questionnaires:  **The modified GHQ-D.** Depression defined as two or more symptoms of depression.  **The Edinburgh Postnatal Depression Scale (EPDS):** A self-administered 10-item questionnaire. A cut-off point of ≥11 was used as an indicator of risk of postnatal depression.  **The Schedules for Clinical Assessment in Neuropsychiatry (SCAN):** A semi-structured clinical interview. Depression was defined according to ICD-10 criteria for depressive disorder.  **Self-efficacy and locus of control:** Measured by three single items. |
| Duffy 1997 | Individually randomized trial conducted in Western Australia. Planned as a pilot study. | 75 primarily low-income women.  Experimental (n=37), control (n=38).  **Inclusion criteria:** primipara women who attended the antenatal clinic of the study hospital; intention to breast feed.  **Exclusion criteria:** delivery before 37 weeks gestation; medical complications. | **Experimental condition:**   - One additional one-hour breast feeding session. - Group size was 6 women. - Session was provided after gestation week 36. - The content of the teaching session was the correct position and attachment of the baby on the breast for feeding. - The instructor was a midwife who was also a senior lactation consultant.   **Control condition:** Standard antenatal education (content not described).The experimental condition received this offer too, | **Obstetric interventions:** Spontaneous vaginal delivery, caesarean section, vacuum extraction, forceps. Measured by questionnaire 24 hours following birth.  **Breast feeding duration:** measured by questionnaire six weeks postnatally. Breast feeding defined as exclusive breast feeding. |
| Feinberg 2008 | Individually randomized trial conducted I USA. | 169 primarily non-Hispanic white couples.  Experimental (n=89), control (n=80).  **Inclusion criteria:** primipara; living together (regardless of marital status). All participants were at least 18 years of age. | **Experimental condition:**   - Psychosocial prevention program for couples with 4 prenatal and 4 postnatal sessions. - Each group consisted of 6–10 couples. - Focus was on emotional self-management, conflict management, problem solving, communication, and mutual support strategies that foster positive joint parenting of an infant. The program was manualized, with didactic material, exercises, and behavioral rehearsal included in the curriculum for each session. - Sessions were led by a male–female team. - No information on how long each session lasted.   **Control condition:** Couples were mailed a brochure about selecting quality child care. | All outcomes were measured by self-reported questionnaires from both mother and father six months postnatally.  **Coparenting:** both parents reported on multiple dimensions (three scales) of the coparenting relationship with a measure developed for the study. All 15 items utilized 7-point Likert response scales.  **Depressive symptoms:** measured with a subset of seven items from the Center for Epidemiological Studies Depression Scale. Items were answered on a 4-point frequency scale.  **Anxiety:** measured with the 20-item short form of the Taylor Manifest Anxiety Scale, which measures chronic anxiety. Items were answered yes/no.  **Parent–Child dysfunctional interaction:** assessed by the 6-item Dysfunctional Interaction Scale from the Parental Stress Index. |
| Forster 2004 | Individually randomized controlled trial with 3 arms: 2 different experimental and 1 control condition.  In this review effects of the most intensive intervention group (attitudes) against the control group is tested. Conducted in Melbourne, Australia. | 654 relatively disadvantaged, low-income women. 92.5 % planned to breast feed.  Experimental (n=327), control (n=327).  **Inclusion criteria:** booking as public patients; primipara; between 16 and 24 weeks pregnant at time of recruitment; and able to speak, read, and write in English.  **Exclusion criteria:** physical problems that prevented breast feeding; and choosing birth center or private obstetric care. | **Experimental condition:**   - Two 1-hour breast feeding education sessions. - Participants were approximately 20 to 25 weeks’ gestation. - Class size of approximately 8 women. Women were encouraged to bring their partners or a significant other. - Sessions focused on changing attitudes to breast feeding, and included information about the advantages of breast feeding, an exploration of the expectant parents’ views and attitudes on breast feeding, and their perceptions of the views of their family and friends, as well as community attitudes, and group discussion. - Sessions were led by midwives and a community educator.   **Control condition:** Standard care including: formal breastfeeding education sessions; lactation consultant  support; community breastfeeding groups; attendance at a breastfeeding information evening; 24-hour telephone counseling support; and a postnatal home visit by a domiciliary midwife. The experimental condition received this offer too, | **Breast feeding initiation:** measured by structured questionnaires by interview 2-4 days postnatally. Defined as breast milk only and any breast milk.  **Breast feeding duration:** measured by telephone interview at six months, postnatally using structured questionnaires. Definitions of breast feeding: breast milk only, any breast milk, and exclusive breast feeding. |
| Ickovics 2007 | A multisite randomized controlled trial was conducted at two university-affiliated hospital prenatal clinics in Connecticut and Atlanta, USA. | 1,047 primarily non-employed African American pregnant women aged 14−25 years.  Experimental (n=653), control (n=394).  **Inclusion criteria:** less than 24 weeks of gestation, age 25 years or less, no medical problems requiring individualized care as “high-risk pregnancy”, English or Spanish language, and willingness to be randomized. | **Experimental condition:**   - General antenatal education. - 10 sessions each lasting 2 hours. - Sessions from gestation week 16-40. - Approximately 8 women in each group. - Content: Group prenatal care across the pregnancy. Focus was on discussion between women and clinicians, and education and skills building to address explicit learning objectives in prenatal care, child birth preparation, and postpartum and parenting roles as well as self-care activities on of weight and blood pressure assessment. - Led by a trained practitioner (midwife or obstetrician).   **Control condition:** Individual prenatal care across the pregnancy occurs over the course of approximately 2 hours in total. | All relevant outcomes were measured during third trimester (on average in gestation week 35).  **Prenatal distress:** measured with the Pregnancy Distress Questionnaire.  **Readiness for labor and delivery:** No description of measurement tool.  **Readiness for infant care:** No description of measurement tool.  **Prenatal knowledge:** measured by a tool developed for the study to assess prenatal and infant care knowledge. |
| Kistin 1990 | Individually randomized trial with 2 arms – ‘breast feeding classes’ and ‘individual sessions’, Conducted in Chicago, USA. | 74 black women born in the US attending a midwife prenatal clinic before their 24^th^ week of gestation.  Experimental (n=38), control (n=36). | **Experimental condition:**   - 50-80 minute breast-feeding class with group discussion. Participants attended at least one (more if they wished). - Topics related to breast feeding/formula use plans, health benefits of breast milk, and common challenges related to breast feeding and how to overcome them. - Sessions led by the authors. - No information on class-sizes or gestation age for education provided.   **Control condition:** One-to-one contact with a medical doctor for 15 to 30 minutes before gestation week 30. The topics discussed were the same as in the experimental group. | **Breast feeding initiation:** measured at an interview in the hospital less than five days postnatally. Breast feeding defined as one or more breast feedings per day.  **Breast feeding duration:** measured by self-reporting. Defined as any breast feeding for 12 weeks or longer. |
| Lara 2010 | Individually randomized trial conducted in Mexico City, Mexico. | 377 low-income pregnant women in high risk of depression.  Experimental (n=250), control (n=127).  **Inclusion criteria:** ≥18 years old, ≤26 weeks pregnant, having completed primary school, did not have any substance abuse, bipolar conditions or reported suicide attempts during the last 6 months, living in the metropolitan area of Mexico City, and meeting criteria for high risk for depression, based on a score of 16 or higher on the Center for Epidemiologic Studies Depression Scale (CES-D) and/or having a self-reported history of depression (only criteria in 43.2 % of the participants).  **Exclusion criteria:** current depression. | **Experimental condition:**   - Psycho-educational program to prevent post-partum depression. - Eight, two-hour weekly sessions. - 5-10 participants per group. - The intervention program had several components: acknowledgement and discussion, as opposed to a formal lecture, of the “normal” perinatal period and risk factors for postpartum depression; increasing positive thinking and pleasant activities; improving self-esteem, and increasing self-care. - Four facilitators delivered the intervention - all of them had extensive clinical experience.   **Control condition:** Standard care as provided by the institutions, including individual prenatal health care (checking for blood pressure, weight check etc.). In some sites they received individual talks on prenatal health care and breathing exercises to use during labor. | **Depressive symptoms:** measured by interview six weeks postnatal with the second edition of the Beck Depression Inventory (BDI-II), a 21 item self-report instrument that explores presence of symptoms during the last two weeks. A cut-off point of ≥14 was used. |
| Le 2011 | Two-cited individually randomized trial conducted in Washington, DC, USA. | 217 predominantly Central American immigrant women in high risk of depression.  Experimental (n=112), control (n=105).  **Inclusion criteria:** 18 –35 years old, ≤24 weeks gestation; no smoking, alcohol, or illicit substance abuse; and at high risk for depression, defined as scoring 16 or higher on the Center for Epidemiological Studies Depression Scale (CES–D) and/or with a self-reported personal or family history of depression.  **Exclusion criteria:** current diagnosis of major depressive disorder, substance abuse, psychosis, a serious medical condition, and/or other significant psychosocial problems. | **Experimental condition:**   - Psycho-educational group sessions. - Eight weekly 2-hour sessions - Content: teaching women mood regulation skills to prevent perinatal depression. - The course was taught in Spanish by one or two postbachelor’s trained bilingual and/or bicultural research staff. - No information on class-sizes or gestation age for education provided.   **Control condition:** Standard care as provided by their prenatal care providers at the clinic. They had the option to participate in group prenatal care classes, if they chose to in addition to their care with a midwife or MD. | **Depressive symptoms:** measured by interview six weeks postnatal with the second edition of the Beck Depression Inventory (BDI-II), a 21 item self-report instrument that explores presence of symptoms during the last two weeks. |
| Maimburg 2010 | Individually randomized controlled trial conducted in Aarhus, Denmark. | 1193 primarily middle-high education level women.  Experimental (n=603), control (n=590).  **Inclusion criteria:** primipara women registered at the Aarhus Midwifery Clinic in Denmark, older than 18 years of age at enrolment, singleton pregnancy, and able to speak and understand Danish. | **Experimental condition:**   - General antenatal education. - Program comprised 3 modules, each lasting 3 hours. - Sessions between 30 and 35 weeks of gestation. - The form was information, video films and group discussions. - Program covered pregnancy issues, birth process, pain delivery, care for the newborn, breast feeding, the transition to parenthood, and postnatal depression. The woman’s partner was also invited to participate - The instructors were midwifes of varying seniority.   **Control condition:** Standard care containing individual consultations with a midwife. No offer on antenatal education but allowed to take other (private) antenatal training. The experimental condition received this offer too, | **Pain relief:** collected from the local hospital database. Overall use of pain relief, overall use of non-pharmacological pain relief, water immersion, acupuncture, intracutaneous sterile water injection, overall use of pharmacological pain relief, nitrous oxide/oxygen, intramuscular morphine, pudendal nerve block, epidural analgesia, other.  **Obstetric interventions:** collected from the local hospital database. Labor induction, oxytocin augmentation, vacuum extraction, caesarean section (elective, emergency).  **Sufficient knowledge about breast feeding:** measured six weeks postnatal by one question developed for the study. Measured on a 5-point Likert scale.  **Breast feeding self-efficacy:** measured with the BSES-SF six weeks postnatally. BSES-SF is a 14-item tool measuring confidence in breast feeding answered on a 5-point Likert scale.  **Breast feeding duration:** measured six weeks and six months postnatal. Breast feeding is both defined as any and exclusive breast feeding.  **Postnatal depression:** measured six weeks postnatal by the self-administered 13-item questionnaire; Edinburgh Postnatal Depression Scale. A cut-off point of ≥12 was used as an indicator of risk of postnatal depression. |
| Maycock 2013 | Individually randomized multicenter trial conducted in 8 hospitals in Perth, Australia. | 1575 (863 women and 712 men) participants were recruited from 8 public hospitals.  No information of number of participants randomized to experimental and control group. The final analysis was completed with 353 women in the experimental and 298 in the control condition.  **Inclusion criteria:** mothers ≥18 years. Fathers had to be contactable by telephone or email, reside within Western Australia; and intend to participate in the rearing of their child. | **Experimental condition:**   - One breast feeding group session for fathers. - 2-hour session with an average group size of 6 fathers. - The main topics of this session were the role of the father, the importance and benefits of breast feeding, and what to expect in the first four weeks at home with a new baby. - From birth and the following 6 weeks, the experimental group of men received written materials aimed to enhance the support for their partner’s breast feeding. - Facilitated by a male instructor.   **Control condition:** Standard care consisting of routine antenatal classes incorporating information on labor, birth, pain relief and breastfeeding. The experimental condition received this offer too, | **Breast feeding:** measured by questionnaire six weeks postnatal. Breast feeding defined as any breast feeding and exclusive breast feeding. |
| Noel-Weiss 2006 | Individually randomized trial conducted in Ontario, Canada. | 101 primarily middle-high SES women. 99 % lived in a supportive relationship and 87 % had made decision to breast feed prior to pregnancy.  No information of number of participants randomized to experimental and control group. The final analysis was completed with 47 women in the experimental and 45 in the control condition.  **Inclusion criteria:** primipara women expecting a single child, an uncomplicated birth, and planning to breastfeed, able to read and write in English and have a telephone to complete the postpartum questionnaires.  **Exclusion criteria:** mother and her infant not discharged at the same time; mother not able to breastfeed without restriction. | **Experimental condition:**   - One prenatal breast feeding workshop lasting 2.5-hour. - Participation of 2-8 women and their partners. - Conducted after gestation week 34. - The form was both based on practical breast feeding exercises with a doll, group discussions, and watching videos with breast feeding. - Facilitated by a registered nurse who had specialized in providing maternity care and breast feeding support. The facilitator was skilled with leading group discussions and providing individual counseling.   **Control condition:** Standard care including the  choice of physician or midwife, frequency of prenatal  visits, and attendance at prenatal classes, was defined by  each mother. The experimental condition received this offer too. | **Maternal breast feeding self-efficacy:** measured with the BSES-SF four and eight weeks postnatal. BSES-SF is a 14-item tool measuring confidence in breast feeding answered on a 5-point Likert scale.  **Breast feeding duration:** measured by asking the mother four and eight weeks postnatal whether she was breast feeding and how much. Breast feeding defined as exclusive breast milk and any breast milk. |
| Ortiz Collado 2014 | A multicentre randomized, longitudinal clinical study conducted in three cities in Spain and France. | 184 primarily low SES women in risk of postnatal depression.  Experimental (n=92), control (n=92).  **Inclusion criteria:** middle or low socio-economic status, <20 weeks of gestation, a moderate to high risk of postnatal depression, no more than two children, no organic serious physical pathology, no psychiatric diagnosis, no alcohol or illicit substance abuse, and able to understand the language.  **Exclusion criteria:** having a current diagnosis of psychiatric disorder or a serious medical condition. | **Experimental condition:**   - Preparation for parenthood group sessions. - 6-8 couples met for 10 weekly sessions each lasting 2 hours and 15 minutes. - Began during the second term of pregnancy. - The classes involved work on individual feelings and affective bonds, with specific objectives for the man and the woman in each participating couple. The program was focusing on preparation for parenting and not just for the childbirth, as well as preparation for both the mother and the father. Each session consisted of an interactive exchange of information (60%) and practical exercises (40%). - No information on educators.   **Control condition:** Standard antenatal education program consisting of eight sessions of two hours each during the third term of pregnancy. The focus was childbirth and pregnancy health. Each group was open and could receive 12 couples or more. | All outcomes were measured by self-reported questionnaires mailed between five and 12 weeks postnatally.  **Depressive symptoms:** Measured by the self-administered 10-item questionnaire; Edinburgh Postnatal Depression Scale.  **Amount of social support received:** Measured by the 11-item Functional Social Support Questionnaire. The questionnaire refers to two dimensions of functional social support: affective support and confidant support. Satisfaction with different situations is rated on a 5-point Likert scale.  **Relationship with partner**: Measured by the 32-item Dyadic Adjustment Scale (DAS). Both mothers and fathers rated items on various Likert-type scales. |
| Rossiter 1994 | Individually randomized trial conducted in Sydney, Australia. | 194 Vietnamese, primarily unemployed, low SES pregnant women.  Experimental (n=108), control (n=86).  **Inclusion criteria:** ethnic Vietnamese or other women who were born and reared in Vietnam; Vietnamese speaking; at least 12 weeks pregnant; gave consent to participate.  **Exclusion criteria:** unforeseen circumstances (miscarriage, stillbirth, change of address). | **Experimental condition:**   - Breast feeding education program. 3 sessions each lasting 2 hours. - Content: a 25-minute videotape followed by small-group discussion sessions. The aims were to provide information on the benefits of breast feeding, relate this information to the women’s background, and discuss any misconceptions about the superiority of formula milk and the norm of infant feeding practices in Australia. - The program was conducted in Vietnamese by the parenthood educators of the hospitals, with the assistance of Vietnamese health interpreter. - No information on class-sizes or gestation age for education provided.   **Control condition:** Participants were provided with official breast feeding and childbirth pamphlets. | **Breast feeding initiation:** measured at visit at the hospital/home visit within one week postnatally. Breast feeding defined as being the main source of nutrition.  **Breast feeding duration:** assessed at home visit six months postnatally. Breast feeding defined as being the main source of nutrition. |
| Rouhe 2013 | Individually randomized trial conducted in Helsinki, Finland. | 371 women with severe fear of childbirth.  Experimental (n=131), control (n=240).  **Inclusion criteria:** Fear of childbirth, defined as a sum score ≥100 on the Wijma Delivery Expectancy Questionnaire; primipara.  **Exclusion criteria:** manifest psychosis; severe depression; serious problems of alcohol or drug abuse. | **Experimental condition:**   - Group psycho-educational classes to reduce fear of birth. - Six 2-hour group sessions during pregnancy from 26^th^ to 35^th^ week gestation and one session 6–8 weeks after delivery. - The focus of the intervention was on increasing individual independence and awareness of one’s own abilities, the choices available during one’s delivery and the successful transition to motherhood. Partners participated in one of the group sessions. - Group size: maximum six women, - Instructor: a psychologist. - No planned visits with an obstetrician.   **Control condition:** A letter in which they were recommended to discuss their fear of childbirth in their primary healthcare maternity unit. Referral to a fear of childbirth team. | All outcomes related to delivery were derived from hospital obstetric patient records.  **Pain relief:** epidural analgesia.  **Obstetric interventions:** Spontaneous vaginal delivery, induction of labor, caesarean section (elective, emergency), vacuum extraction. |
| Schulz 2006 | Individually randomized trial conducted in California, USA. | 52 primarily European American couples.  Experimental (n=28 couples), control (n=24 couples).  **Inclusion criteria:** couples living together, expecting their first child, and over 18 years of age. | **Experimental condition**:   - Couple-focused intervention for partners becoming parents. - 24 weekly 2.5 hour couple group sessions. - Sessions from 3 months before birth - 3 months after birth. - Each group included 4 couples and one co-leader married couple. - Topics for discussion included: how participants viewed themselves and their relationships, division of family labor, communication and problem-solving styles, and relationship as a couple.   **Control condition:** Standard care, including home and lab visits (included interviews, interaction tasks, and cognitive assessments of the children after birth), The experimental condition received this offer too. | **Marital satisfaction:** measured by the MAT questionnaire containing 16 items. Both men and women answered this questionnaire at six months and 66 months postnatally.  **Divorce/separation:** reported at 66 months postnatally. |
| Werner 2013 | Individually randomized single-blind controlled trial with 3 arms: one experimental group, an active comparison group and one control group. In this review effects of the most intensive group-based program (hypnosis) against the control group is tested. Conducted in Aarhus, Denmark. | 727 women  Experimental (n=497), control (n = 230).  **Inclusion criteria:** no chronic diseases, uncomplicated pregnancy, primipara, older than 18 years, and able to understand and speak Danish. | **Experimental condition:**   - Self-hypnosis for childbirth-education. - Three 1-hour sessions held over three consecutive weeks. The first session lasted 2.5 hours. - The program included three audiorecordings including a 20-minute section especially meant for labor. - Classes were taught by midwifes trained in hypnosis. - No information on group size provided.   **Control condition:** Standard care consisting of 4-5 individual consultations with a midwife and a tour of the birth department. The experimental condition received this offer too. | All outcomes related to delivery were extracted from an ongoing data collection from all births at the hospital or from medical records.  **Pain relief:** use of epidural analgesia during birth.  **Obstetric interventions:** spontaneous vaginal birth, cesarean section (elective, emergency), oxytocin augmentation, vacuum extraction.  **Breast feeding duration (any breast feeding):** derived from questionnaires four months postnatally. |
| Westney 1988 | Individually randomized trial. | A volunteer sample of 28 black, unmarried, 15-18 year old prospective fathers.  Experimental (n=15), control (n=13). | **Experimental condition:**   - Prenatal classes 4 times weekly each lasting 2 hours. - Classes addressed human sexuality, pregnancy and prenatal care, labor and delivery, infant and child care. - Teaching approaches included lectures, audiovisual aids, and group discussions of concerns. - Presented by a female registered nurse-specialist in maternal-child care. - No information on class-sizes or gestation age for education provided.   **Control condition:** Standard care (content not described). No participants reported to participate in any other pregnancy-related education program. | **Paternal knowledge of human sexuality, pregnancy and prenatal care, labor and delivery, infant and child care, and support towards the mother:** measured after last experimental session (gestation week unknown) using a 75-item questionnaire developed for the study. |

Brugha TS, Wheatley S, Taub NA, Culverwell A, Friedman T, Kirwan P, Jones DR, Shapiro DA: **Pragmatic randomized trial of antenatal intervention to prevent post-natal depression by reducing psychosocial risk factors.** *Psychological medicine* 2000, **30:**1273-1281.

Duffy EP, Percival P, Kershaw E: **Positive effects of an antenatal group teaching session on postnatal nipple pain, nipple trauma and breast feeding rates.** *Midwifery* 1997, **13:**189-196.

Feinberg ME, Kan ML: **Establishing family foundations: intervention effects on coparenting, parent/infant well-being, and parent-child relations.** *Journal of family psychology : JFP : journal of the Division of Family Psychology of the American Psychological Association (Division 43)* 2008, **22:**253-263.

Forster D, McLachlan H, Lumley J, Beanland C, Waldenstrom U, Amir L: **Two mid-pregnancy interventions to increase the initiation and duration of breastfeeding: a randomized controlled trial.** *Birth (Berkeley, Calif)* 2004, **31:**176-182.

Ickovics JR, Kershaw TS, Westdahl C, Magriples U, Massey Z, Reynolds H, Rising SS: **Group prenatal care and perinatal outcomes: a randomized controlled trial.** *Obstetrics and gynecology* 2007, **110:**330-339.

Kistin N, Benton D, Rao S, Sullivan M: **Breast-feeding rates among black urban low-income women: effect of prenatal education.** *Pediatrics* 1990, **86:**741-746.

Lara MA, Navarro C, Navarrete L: **Outcome results of a psycho-educational intervention in pregnancy to prevent PPD: a randomized control trial.** *Journal of affective disorders* 2010, **122:**109-117.

Le HN, Perry DF, Stuart EA: **Randomized controlled trial of a preventive intervention for perinatal depression in high-risk Latinas.** *Journal of consulting and clinical psychology* 2011, **79:**135-141.

Maimburg RD, Vaeth M, Durr J, Hvidman L, Olsen J: **Randomised trial of structured antenatal training sessions to improve the birth process.** *BJOG : an international journal of obstetrics and gynaecology* 2010, **117:**921-928.

Maycock B, Binns CW, Dhaliwal S, Tohotoa J, Hauck Y, Burns S, Howat P: **Education and support for fathers improves breastfeeding rates: a randomized controlled trial.** *Journal of human lactation : official journal of International Lactation Consultant Association* 2013, **29:**484-490.

Noel-Weiss J, Rupp A, Cragg B, Bassett V, Woodend AK: **Randomized controlled trial to determine effects of prenatal breastfeeding workshop on maternal breastfeeding self-efficacy and breastfeeding duration.** *Journal of obstetric, gynecologic, and neonatal nursing : JOGNN / NAACOG* 2006, **35:**616-624.

Ortiz Collado MA, Saez M, Favrod J, Hatem M: **Antenatal psychosomatic programming to reduce postpartum depression risk and improve childbirth outcomes: a randomized controlled trial in Spain and France.** *BMC pregnancy and childbirth* 2014, **14:**22.

Rossiter JC: **The effect of a culture-specific education program to promote breastfeeding among Vietnamese women in Sydney.** *International*

*journal of nursing studies* 1994, **31:**369-379.

Rouhe H, Salmela-Aro K, Toivanen R, Tokola M, Halmesmaki E, Saisto T: **Obstetric outcome after intervention for severe fear of childbirth in nulliparous women - randomised trial.** *BJOG : an international journal of obstetrics and gynaecology* 2013, **120:**75-84.

Schulz MS, Cowan CP, Cowan PA: **Promoting healthy beginnings: a randomized controlled trial of a preventive intervention to preserve marital quality during the transition to parenthood.** *Journal of consulting and clinical psychology* 2006, **74:**20-31.

Werner A, Uldbjerg N, Zachariae R, Rosen G, Nohr EA: **Self-hypnosis for coping with labour pain: a randomised controlled trial.** *BJOG : an international journal of obstetrics and gynaecology* 2013, **120:**346-353.

Westney OE, Cole OJ, Munford TL: **The effects of prenatal education intervention on unwed prospective adolescent fathers.** *Journal of adolescent health care : official publication of the Society for Adolescent Medicine* 1988, **9:**214-218.
