# Supplementary material for: The effect of antenatal education in small classes on obstetric and psycho-social outcomes - a systematic review
Source: Syst Rev. 2015 Feb 28;4:20. doi: 10.1186/s13643-015-0010-x (PMC4355374; doi:10.1186/s13643-015-0010-x)
Supplement: Additional file 6: — Effect tables. The file contains tables of measures of intervention effects (RR and MD) with 95% confidence intervals and two-sided P values for each outcome in the included trials. [file 13643_2015_10_MOESM6_ESM.docx]

**Effect tables**

**Comparison 1: Depression prevention classes versus standard care**

| **Outcome or Subgroup** | **Studies** | **Participants** | **Statistical Method** | **Effect Estimate** |
| --- | --- | --- | --- | --- |
| 1.1 Depression 3 months postnatal (EPDS) | 1 | 190 | Risk Ratio (M-H, Fixed, 95% CI) | 0.85 [0.46, 1.59] |
| 1.2 Depression 3 months postnatal (GHQ-D tool) | 1 | 190 | Risk Ratio (M-H, Fixed, 95% CI) | 1.17 [0.70, 1.95] |
| 1.3 Depression 3 months postnatal (Scan tool) | 1 | 190 | Risk Ratio (M-H, Fixed, 95% CI) | 0.51 [0.13, 1.98] |
| 1.4 Depressive symptoms in pregnancy (BDI-II tool) | 1 | 186 | Mean Difference (IV, Fixed, 95% CI) | -2.10 [-4.61, 0.41] |
| 1.5 Depressive symptoms 6 weeks postnatal (BDI-II tool) | 1 | 180 | Mean Difference (IV, Fixed, 95% CI) | 0.31 [-2.10, 2.72] |
| 1.6 Depressive symptoms 6 weeks postnatal (> 14 on BDI-II tool) | 1 | 149 | Risk Ratio (M-H, Fixed, 95% CI) | 0.95 [0.62, 1.45] |
| 1.7 High vs. low confidence in ability to solve problems 3 months postnatal | 1 | 190 | Risk Ratio (M-H, Fixed, 95% CI) | 0.71 [0.28, 1.80] |
| 1.8 High vs. low belief in personal control when solving problems 3 months postnatal | 1 | 190 | Risk Ratio (M-H, Fixed, 95% CI) | 1.07 [0.80, 1.43] |
| 1.9 High vs. low belief in internal factors influencing their life 3 months postnatal | 1 | 190 | Risk Ratio (M-H, Fixed, 95% CI) | 0.98 [0.73, 1.31] |

**Comparison 2: Psycho-social prevention program versus brochure**

| **Outcome or Subgroup** | **Studies** | **Participants** | **Statistical Method** | **Effect Estimate** |
| --- | --- | --- | --- | --- |
| 2.1 Depressive symptoms women 6 months postnatal (CESDS) | 1 | 152 | Mean Difference (IV, Fixed, 95% CI) | -0.08 [-0.21, 0.05] |
| 2.2 Depressive symptoms men 6 months postnatal (CESDS) | 1 | 152 | Mean Difference (IV, Fixed, 95% CI) | 0.02 [-0.07, 0.11] |
| 2.3 Co-parental support women 6 months postnatal | 1 | 152 | Mean Difference (IV, Fixed, 95% CI) | 0.30 [-0.04, 0.64] |
| 2.4 Co-parental support men 6 months postnatal | 1 | 152 | Mean Difference (IV, Fixed, 95% CI) | 0.29 [0.05, 0.53] |
| 2.5 Co-parental undermining women 6 months postnatal | 1 | 152 | Mean Difference (IV, Fixed, 95% CI) | 0.04 [-0.20, 0.28] |
| 2.6 Co-parental undermining men 6 months postnatal | 1 | 152 | Mean Difference (IV, Fixed, 95% CI) | 0.10 [-0.16, 0.36] |
| 2.7 Parenting-based closeness women 6 months postnatal | 1 | 152 | Mean Difference (IV, Fixed, 95% CI) | 0.06 [-0.30, 0.42] |
| 2.8 Parenting-based closeness men 6 months postnatal | 1 | 152 | Mean Difference (IV, Fixed, 95% CI) | 0.35 [0.04, 0.66] |
| 2.9 Anxiety women 6 months postnatal | 1 | 152 | Mean Difference (IV, Fixed, 95% CI) | -0.11 [-1.53, 1.31] |
| 2.10 Anxiety men 6 months postnatal | 1 | 152 | Mean Difference (IV, Fixed, 95% CI) | -0.79 [-2.02, 0.44] |
| 2.11 Mother-child dysfunctional interaction 6 months postnatal | 1 | 152 | Mean Difference (IV, Fixed, 95% CI) | -0.10 [-0.25, 0.05] |
| 2.12 Father-child dysfunctional interaction 6 months postnatal | 1 | 152 | Mean Difference (IV, Fixed, 95% CI) | -0.26 [-0.43, -0.09] |

**Comparison 3: Psycho-educational classes versus letter**

| **Outcome or Subgroup** | **Studies** | **Participants** | **Statistical Method** | **Effect Estimate** |
| --- | --- | --- | --- | --- |
| 3.1 Epidural analgesia | 1 | 371 | Risk Ratio (M-H, Fixed, 95% CI) | 1.13 [0.96, 1.33] |
| 3.2 Spontaneous delivery | 1 | 371 | Risk Ratio (M-H, Fixed, 95% CI) | 1.33 [1.11, 1.61] |
| 3.3 Overall caesarean section | 1 | 371 | Risk Ratio (M-H, Fixed, 95% CI) | 0.70 [0.49, 1.01] |
| 3.4 Elective caesarean section | 1 | 371 | Risk Ratio (M-H, Fixed, 95% CI) | 0.83 [0.46, 1.50] |
| 3.5 Emergency caesarean section | 1 | 371 | Risk Ratio (M-H, Fixed, 95% CI) | 0.62 [0.37, 1.06] |
| 3.6 Vacuum extraction | 1 | 371 | Risk Ratio (M-H, Fixed, 95% CI) | 0.69 [0.42, 1.13] |
| 3.7 Induction of labor | 1 | 371 | Risk Ratio (M-H, Fixed, 95% CI) | 1.10 [0.74, 1.64] |

**Comparison 4: Program with psycho-somatic approach versus standard antenatal education program**

| **Outcome or Subgroup** | **Studies** | **Participants** | **Statistical Method** | **Effect Estimate** |
| --- | --- | --- | --- | --- |
| 4.1 Depressive symptoms 5-12 weeks postnatal (EPDS) | 1 | 127 | Mean Difference (IV, Fixed, 95% CI) | -1.77 [-3.75, 0.21] |
| 4.2 Lack of social support 5-12 weeks postnatal(Functional Social Support Questionnaire) | 1 | 127 | Mean Difference (IV, Fixed, 95% CI) | -1.61 [-4.66, 1.44] |
| 4.3 Dissatisfaction with relationship women 5-12 weeks postnatal (DASS) | 1 | 127 | Mean Difference (IV, Fixed, 95% CI) | 5.38 [-4.07, 14.83] |
| 4.4 Dissatisfaction with relationship men 5-12 weeks postnatal (DASS) | 1 | 127 | Mean Difference (IV, Fixed, 95% CI) | 4.30 [-1.21, 9.81] |

**Comparison 5: Couple-focused classes versus standard care**

| **Outcome or Subgroup** | **Studies** | **Participants** | **Statistical Method** | **Effect Estimate** |
| --- | --- | --- | --- | --- |
| 5.1 Marital satisfaction women 6 months postnatal | 1 | 49 | Mean Difference (IV, Fixed, 95% CI) | -5.54 [-16.95, 5.87] |
| 5.2 Marital satisfaction men 6 months postnatal | 1 | 49 | Mean Difference (IV, Fixed, 95% CI) | -0.50 [-9.77, 8.77] |
| 5.3 Marital satisfaction women 5.5 years postnatal | 1 | 29 | Mean Difference (IV, Fixed, 95% CI) | 8.90 [-10.47, 28.27] |
| 5.4 Marital satisfaction men 5.5 years postnatal | 1 | 29 | Mean Difference (IV, Fixed, 95% CI) | 5.33 [-9.58, 20.24] |
| 5.5 Divorce/separation 5.5 years postnatal | 1 | 45 | Risk Ratio (M-H, Fixed, 95% CI) | 1.20 [0.37, 3.88] |

**Comparison 6: Self-hypnosis classes versus standard care**

| **Outcome or Subgroup** | **Studies** | **Participants** | **Statistical Method** | **Effect Estimate** |
| --- | --- | --- | --- | --- |
| 6.1 Epidural analgesia | 1 | 723 | Risk Ratio (M-H, Fixed, 95% CI) | 1.04 [0.82, 1.32] |
| 6.2 Spontaneous delivery | 1 | 723 | Risk Ratio (M-H, Fixed, 95% CI) | 1.00 [0.90, 1.11] |
| 6.3 Overall caesarean section | 1 | 723 | Risk Ratio (M-H, Fixed, 95% CI) | 1.25 [0.89, 1.76] |
| 6.4 Elective caesarean section | 1 | 723 | Risk Ratio (M-H, Fixed, 95% CI) | 0.51 [0.22, 1.19] |
| 6.5 Emergency caesarean section | 1 | 723 | Risk Ratio (M-H, Fixed, 95% CI) | 1.52 [1.02, 2.27] |
| 6.6 Vacuum extraction | 1 | 723 | Risk Ratio (M-H, Fixed, 95% CI) | 0.75 [0.51, 1.10] |
| 6.7 Oxytocin augmentation | 1 | 723 | Risk Ratio (M-H, Fixed, 95% CI) | 1.09 [0.90, 1.30] |
| 6.8 Labor induction | 1 | 723 | Risk Ratio (M-H, Fixed, 95% CI) | 0.91 [0.79, 1.04] |
| 6.9 Any breast feeding 4 months postnatal | 1 | 698 | Risk Ratio (M-H, Fixed, 95% CI) | 0.97 [0.91, 1.04] |

**Comparison 7: General antenatal education classes versus standard care**

| **Outcome or Subgroup** | **Studies** | **Participants** | **Statistical Method** | **Effect Estimate** |
| --- | --- | --- | --- | --- |
| 7.1 Overall pain relief | 1 | 1162 | Risk Ratio (M-H, Fixed, 95% CI) | 1.00 [0.94, 1.05] |
| 7.2 Overall pharmacological pain relief | 1 | 1162 | Risk Ratio (M-H, Fixed, 95% CI) | 0.93 [0.86, 1.01] |
| 7.3 Epidural analgesia | 1 | 1162 | Risk Ratio (M-H, Fixed, 95% CI) | 0.84 [0.73, 0.98] |
| 7.4 Nitrous oxide/oxygen | 1 | 1162 | Risk Ratio (M-H, Fixed, 95% CI) | 1.03 [0.90, 1.19] |
| 7.5 Intramuscular morphine | 1 | 1162 | Risk Ratio (M-H, Fixed, 95% CI) | 0.68 [0.29, 1.57] |
| 7.6 Pudendal nerve block | 1 | 1162 | Risk Ratio (M-H, Fixed, 95% CI) | 1.27 [0.78, 2.07] |
| 7.7 Other pharmacological (primary halcion, codein, paracetamol) | 1 | 1162 | Risk Ratio (M-H, Fixed, 95% CI) | 0.89 [0.63, 1.24] |
| 7.8 Overall non-pharmacological pain relief | 1 | 1162 | Risk Ratio (M-H, Fixed, 95% CI) | 1.04 [0.94, 1.15] |
| 7.9 Water immersion | 1 | 1162 | Risk Ratio (M-H, Fixed, 95% CI) | 1.00 [0.87, 1.16] |
| 7.10 Acupuncture | 1 | 1162 | Risk Ratio (M-H, Fixed, 95% CI) | 0.94 [0.79, 1.14] |
| 7.11 Intracutaneous sterile water injection | 1 | 1162 | Risk Ratio (M-H, Fixed, 95% CI) | 0.88 [0.67, 1.16] |
| 7.12 Spontaneous delivery | 1 | 1162 | Risk Ratio (M-H, Fixed, 95% CI) | 1.03 [0.94, 1.13] |
| 7.13 Overall caesarean section | 1 | 1162 | Risk Ratio (M-H, Fixed, 95% CI) | 0.91 [0.73, 1.15] |
| 7.14 Elective caesarean section | 1 | 1162 | Risk Ratio (M-H, Fixed, 95% CI) | 0.98 [0.57, 1.68] |
| 7.15 Emergency caesarean section | 1 | 1162 | Risk Ratio (M-H, Fixed, 95% CI) | 0.90 [0.69, 1.17] |
| 7.16 Vacuum extraction | 1 | 1162 | Risk Ratio (M-H, Fixed, 95% CI) | 1.03 [0.80, 1.33] |
| 7.17 Oxytocin augmentation | 1 | 1162 | Risk Ratio (M-H, Fixed, 95% CI) | 0.97 [0.87, 1.08] |
| 7.18 Labor induction | 1 | 1162 | Risk Ratio (M-H, Fixed, 95% CI) | 1.02 [0.91, 1.15] |
| 7.19 Sufficient knowledge about breast feeding | 1 | 1060 | Risk Ratio (M-H, Fixed, 95% CI) | 1.08 [1.01, 1.15] |
| 7.20 Exclusive breast feeding 6 weeks postnatal | 1 | 1048 | Risk Ratio (M-H, Fixed, 95% CI) | 1.01 [0.95, 1.07] |
| 7.21 Any breast feeding 6 weeks postnatal | 1 | 836 | Risk Ratio (M-H, Fixed, 95% CI) | 1.01 [0.98, 1.04] |
| 7.22 Exclusive breast feeding 6 months postnatal | 1 | 1048 | Risk Ratio (M-H, Fixed, 95% CI) | 0.88 [0.55, 1.41] |
| 7.23 Any breast feeding 6 months postnatal | 1 | 836 | Risk Ratio (M-H, Fixed, 95% CI) | 1.02 [0.92, 1.12] |
| 7.24 Postnatal depression 6 weeks postnatal (EPDS) | 1 | 1069 | Risk Ratio (M-H, Fixed, 95% CI) | 0.90 [0.59, 1.37] |
| 7.25 Breast feeding self-efficacy 6 weeks postnatal | 1 | 1058 | Mean Difference (IV, Fixed, 95% CI) | 0.03 [-0.05, 0.11] |

**Comparison 8: Group prenatal care (20 hours) versus individual prenatal care (2 hours)**

| **Outcome or Subgroup** | **Studies** | **Participants** | **Statistical Method** | **Effect Estimate** |
| --- | --- | --- | --- | --- |
| 8.1 Prenatal and infant care knowledge | 1 | 934 | Mean Difference (IV, Fixed, 95% CI) | 2.60 [1.68, 3.52] |
| 8.2 Readiness for labor and delivery | 1 | 934 | Mean Difference (IV, Fixed, 95% CI) | 7.60 [3.34, 11.86] |
| 8.3 Readiness for infant care | 1 | 934 | Mean Difference (IV, Fixed, 95% CI) | 3.10 [-0.14, 6.34] |
| 8.4 Prenatal distress | 1 | 934 | Mean Difference (IV, Fixed, 95% CI) | -0.40 [-1.33, 0.53] |

**Comparison 9: Paternal education class versus standard care**

| **Outcome or Subgroup** | **Studies** | **Participants** | **Statistical Method** | **Effect Estimate** |
| --- | --- | --- | --- | --- |
| 9.1 Paternal knowledge | 1 | 28 | Mean Difference (IV, Fixed, 95% CI) | 9.55 [1.25, 17.85] |
| 9.2 Exclusive breast feeding 6 weeks postnatal | 1 | 651 | Risk Ratio (M-H, Fixed, 95% CI) | 1.04 [0.88, 1.23] |
| 9.3 Any breast feeding 6 weeks postnatal | 1 | 651 | Risk Ratio (M-H, Fixed, 95% CI) | 1.09 [1.00, 1.18] |

**Comparison 10: Extra breast feeding sessions versus standard care**

| **Outcome or Subgroup** | **Studies** | **Participants** | **Statistical Method** | **Effect Estimate** |
| --- | --- | --- | --- | --- |
| 10.1 Spontaneous delivery | 1 | 70 | Risk Ratio (M-H, Fixed, 95% CI) | 1.04 [0.77, 1.42] |
| 10.2 Overall caesarean section | 1 | 70 | Risk Ratio (M-H, Fixed, 95% CI) | 0.67 [0.12, 3.75] |
| 10.3 Vacuum extraction | 1 | 70 | Risk Ratio (M-H, Fixed, 95% CI) | 1.00 [0.36, 2.80] |
| 10.4 Forceps | 1 | 70 | Risk Ratio (M-H, Fixed, 95% CI) | 1.00 [0.15, 6.71] |
| 10.5 Breast feeding initiation – breast milk only | 1 | 618 | Risk Ratio (M-H, Fixed, 95% CI) | 0.99 [0.91, 1.08] |
| 10.6 Breast feeding initiation – any breast milk | 1 | 618 | Risk Ratio (M-H, Fixed, 95% CI) | 0.99 [0.95, 1.02] |
| 10.7 Exclusive breast feeding 6 weeks postnatal | 1 | 70 | Risk Ratio (M-H, Fixed, 95% CI) | 3.20 [1.88, 5.46] |
| 10.8 Breast milk only 8 weeks postnatal | 1 | 92 | Risk Ratio (M-H, Fixed, 95% CI) | 1.12 [0.85, 1.49] |
| 10.9 Any breast milk 8 weeks postnatal | 1 | 92 | Risk Ratio (M-H, Fixed, 95% CI) | 0.96 [0.33, 2.75] |
| 10.10 Exclusive breast feeding 6 months postnatal | 1 | 592 | Risk Ratio (M-H, Fixed, 95% CI) | 1.16 [0.67, 2.01] |
| 10.11 Breast milk only 6 months postnatal | 1 | 592 | Risk Ratio (M-H, Fixed, 95% CI) | 0.96 [0.77, 1.20] |
| 10.12 Any breast milk 6 months postnatal | 1 | 592 | Risk Ratio (M-H, Fixed, 95% CI) | 0.92 [0.79, 1.07] |
| 10.13 Breast feeding self-efficacy 4 weeks postnatal | 1 | 80 | Mean Difference (IV, Fixed, 95% CI) | 4.60 [0.72, 8.48] |
| 10.14 Breast feeding self-efficacy 8 weeks postnatal | 1 | 74 | Mean Difference (IV, Fixed, 95% CI) | 2.79 [-0.76, 6.34] |

**Comparison 11: Breast feeding classes versus one-to-one contact**

| **Outcome or Subgroup** | **Studies** | **Participants** | **Statistical Method** | **Effect Estimate** |
| --- | --- | --- | --- | --- |
| 11.1 Breast feeding initiation - one or more breast feedings per day | 1 | 74 | Risk Ratio (M-H, Fixed, 95% CI) | 0.89 [0.55, 1.45] |
| 11.2 Any breast feeding 12 weeks postnatal | 1 | 74 | Risk Ratio (M-H, Fixed, 95% CI) | 2.84 [0.61, 13.18] |

**Comparison 12: Breast feeding classes versus breast feeding and childbirth pamphlets**

| **Outcome or Subgroup** | **Studies** | **Participants** | **Statistical Method** | **Effect Estimate** |
| --- | --- | --- | --- | --- |
| 12.1 Breast feeding initiation - main source of nutrition | 1 | 178 | Risk Ratio (M-H, Fixed, 95% CI) | 1.86 [1.35, 2.55] |
| 12.2 Breast feeding as main source of nutrition 6 months postnatal | 1 | 175 | Risk Ratio (M-H, Fixed, 95% CI) | 1.59 [0.86, 2.94] |
