# Supplementary material for: The effect of antenatal education in small classes on obstetric and psycho-social outcomes - a systematic review
Source: Syst Rev. 2015 Feb 28;4:20. doi: 10.1186/s13643-015-0010-x (PMC4355374; doi:10.1186/s13643-015-0010-x)
Supplement: Additional file 7: — Forest plots. The file contains forest plots of intervention effects (RR and MD) for each outcome in the included trials. [file 13643_2015_10_MOESM7_ESM.docx]

**Forest plots**

Comparison 1: Depression prevention versus standard care, outcome 1: Depression 3 months postnatal (EPDS tool)


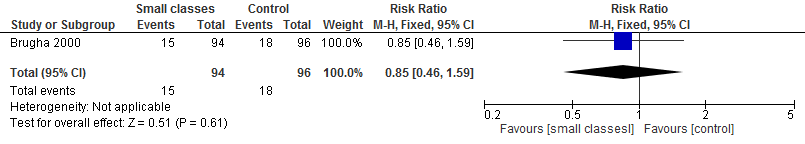


Comparison 1: Depression prevention versus standard care, outcome 2: Depression 3 months postnatal (GHQ-D tool)


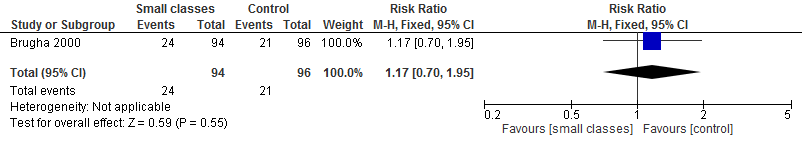


Comparison 1: Depression prevention versus standard care, outcome 3: Depression 3 months postnatal (SCAN tool)


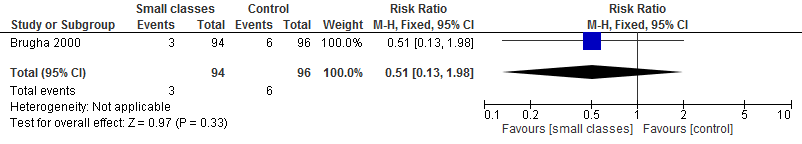


Comparison 1: Depression prevention versus standard care, outcome 4: Depressive symptoms in pregnancy (BDI-II tool)


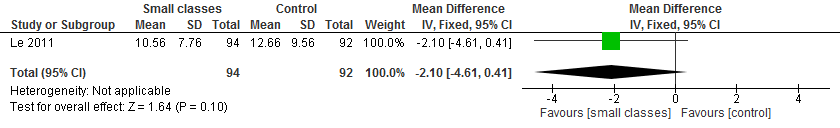


Comparison 1: Depression prevention versus standard care, outcome 5: Depressive symptoms 6 weeks postnatal (BDI-II tool)


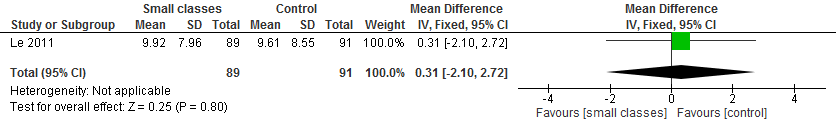


Comparison 1: Depression prevention versus standard care, outcome 6: Depressive symptoms 6 weeks postnatal (> 14 on BDI-II tool)


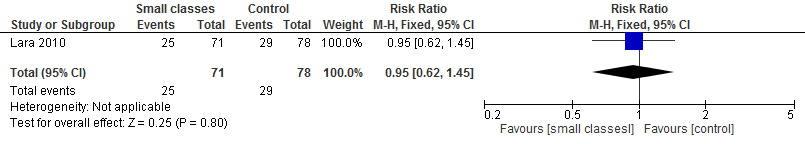


Comparison 1: Depression prevention versus standard care, outcome 7: High vs. low confidence in ability to solve problems 3 months postnatal


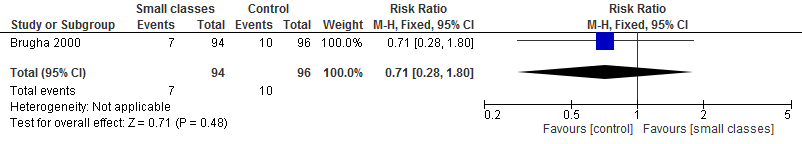


Comparison 1: Depression prevention versus standard care, outcome 8: High vs. low belief in personal control when solving problems 3 months postnatal


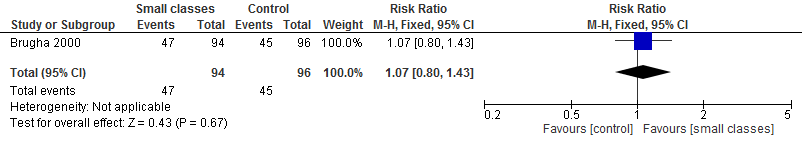


Comparison 1: Depression prevention versus standard care, outcome 9: High vs. low belief in internal factors influencing their life 3 months postnatal


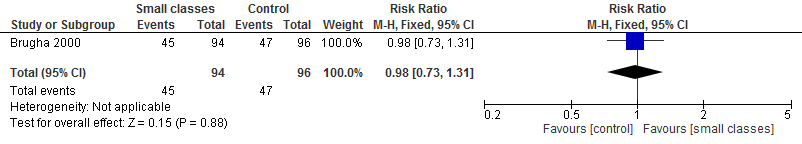


Comparison 2: Psycho-social prevention program versus brochure on child care, outcome 1: Depressive symptoms women 6 months postnatal (CESDS)


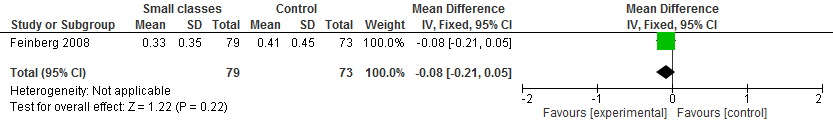


Comparison 2: Psycho-social prevention program versus brochure on child care, outcome 2: Depressive symptoms men 6 months postnatal (CESDS)


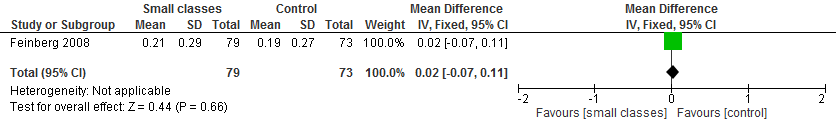


Comparison 2: Psycho-social prevention program versus brochure on child care, outcome 3: Co-parental support women 6 months postnatal


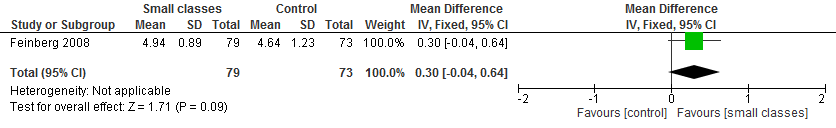


Comparison 2: Psycho-social prevention program versus brochure on child care, outcome 4: Co-parental support men 6 months postnatal


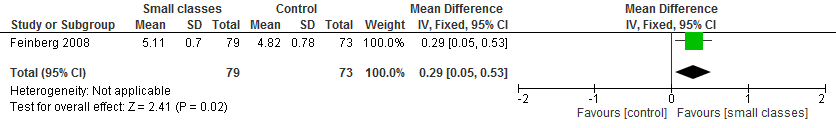


Comparison 2: Psycho-social prevention program versus brochure on child care, outcome 5: Co-parental undermining women 6 months postnatal


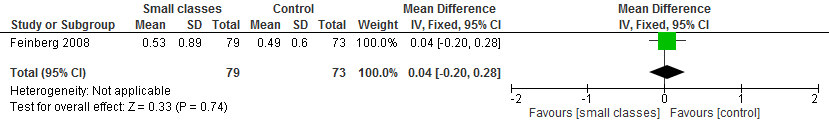


Comparison 2: Psycho-social prevention program versus brochure on child care, outcome 6: Co-parental undermining men 6 months postnatal


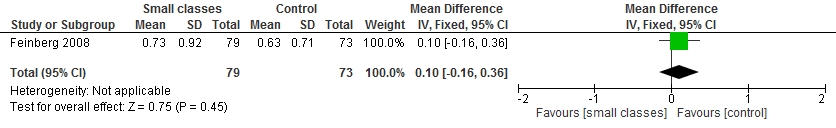


Comparison 2: Psycho-social prevention program versus brochure on child care, outcome 7: Parenting-based closeness women 6 months postnatal


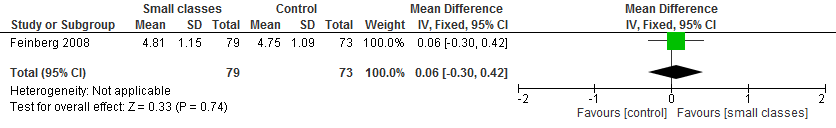


Comparison 2: Psycho-social prevention program versus brochure on child care, outcome 8: Parenting-based closeness men 6 months postnatal


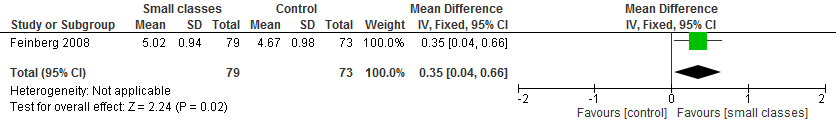


Comparison 2: Psycho-social prevention program versus brochure on child care, outcome 9: Anxiety women 6 months postnatal


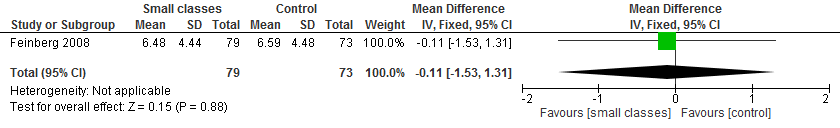


Comparison 2: Psycho-social prevention program versus brochure on child care, outcome 10: Anxiety men 6 months postnatal


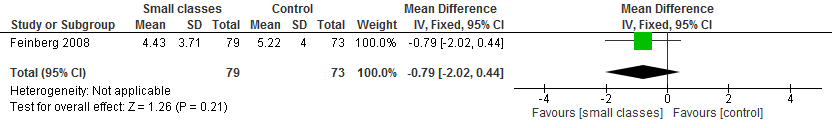


Comparison 2: Psycho-social prevention program versus brochure on child care, outcome 11: Mother-child dysfunctional interaction 6 months postnatal


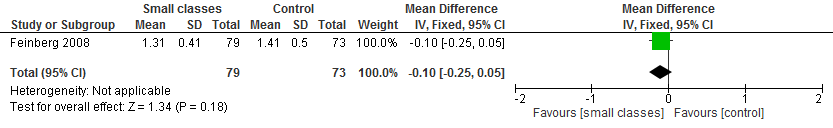


Comparison 2: Psycho-social prevention program versus brochure on child care, outcome 12: Father-child dysfunctional interaction 6 months postnatal


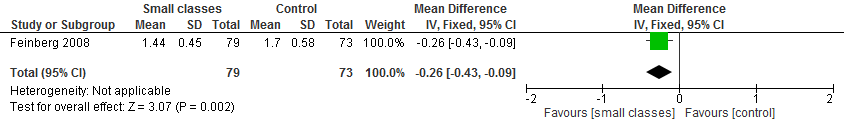


Comparison 3: Psycho-educational classes versus letter on fear of childbirth, outcome 1: Epidural analgesia


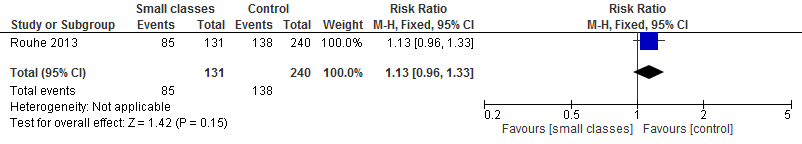


Comparison 3: Psycho-educational classes versus letter on fear of childbirth, outcome 2: Spontaneous delivery


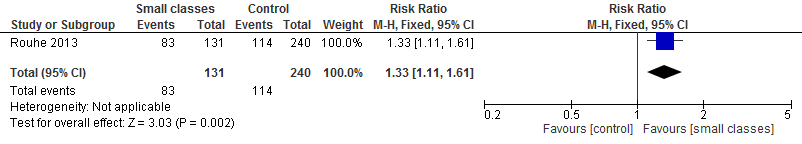


Comparison 3: Psycho-educational classes versus letter on fear of childbirth, outcome 3: Overall caesarean section


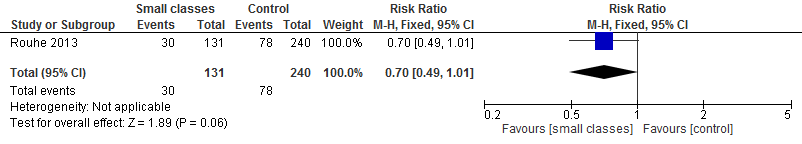


Comparison 3: Psycho-educational classes versus letter on fear of childbirth, outcome 4: Elective caesarean section


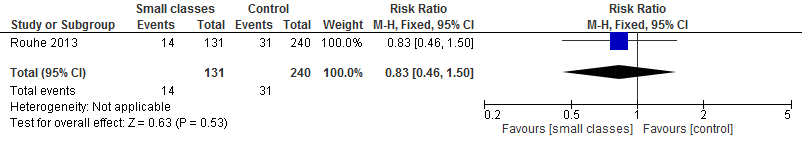


Comparison 3: Psycho-educational classes versus letter on fear of childbirth, outcome 5: Emergency caesarean section


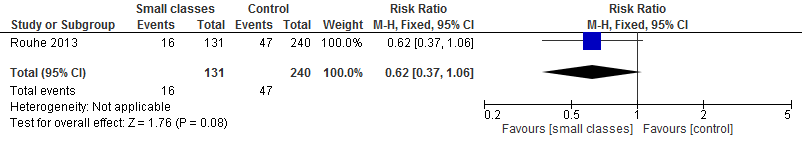


Comparison 3: Psycho-educational classes versus letter on fear of childbirth, outcome 6: Vacuum extraction


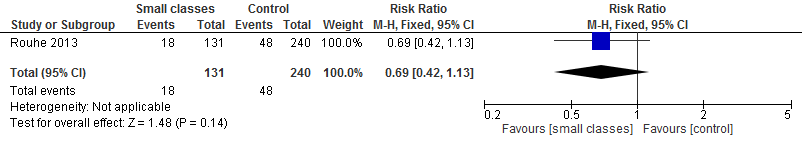


Comparison 3: Psycho-educational classes versus letter on fear of childbirth, outcome 7: Induction of labor


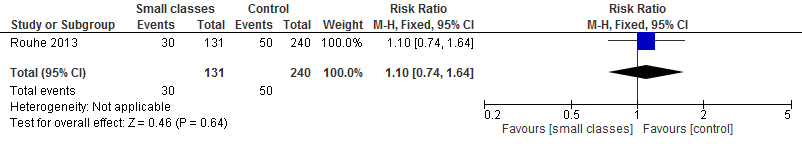


Comparison 4: Program with psycho-somatic approach versus standard antenatal education program, outcome 1: Depressive symptoms 5-12 weeks postnatal (EPDS)


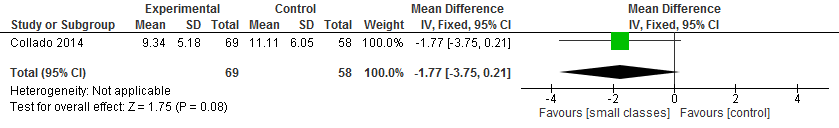


Comparison 4: Program with psycho-somatic approach versus standard antenatal education program, outcome 2: Lack of social support 5-12 weeks postnatal (Functional Social Support Questionnaire)


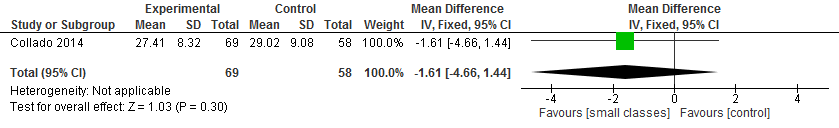


Comparison 4: Program with psycho-somatic approach versus standard antenatal education program, outcome 3: Dissatisfaction with relationship women 5-12 weeks postnatal (DASS)


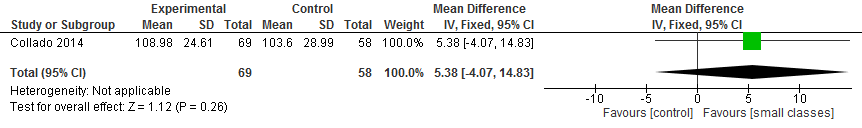


Comparison 4: Program with psycho-somatic approach versus standard antenatal education program, outcome 4: Dissatisfaction with relationship men 5-12 weeks postnatal (DASS)


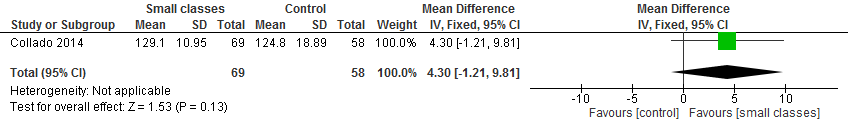


Comparison 5: Couple-focused classes versus standard care, outcome 1: Marital satisfaction women 6 months postnatal


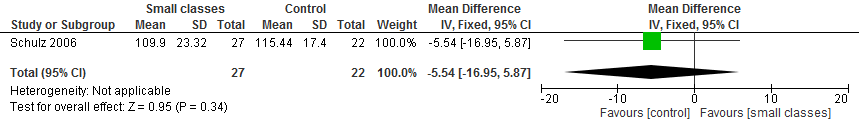


Comparison 5: Couple-focused classes versus standard care, outcome 2: Marital satisfaction men 6 months postnatal


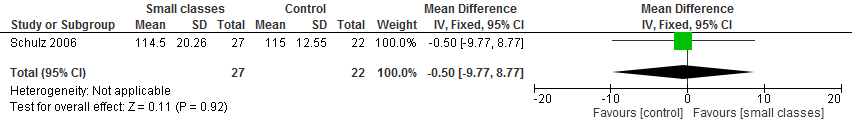


Comparison 5: Couple-focused classes versus standard care, outcome 3: Marital satisfaction women 5.5 years postnatal


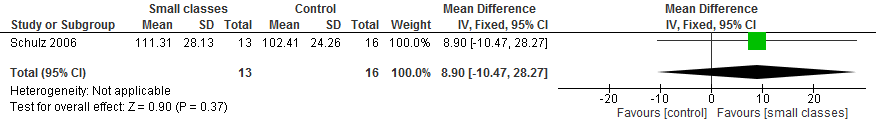


Comparison 5: Couple-focused classes versus standard care, outcome 4: Marital satisfaction men 5.5 years postnatal


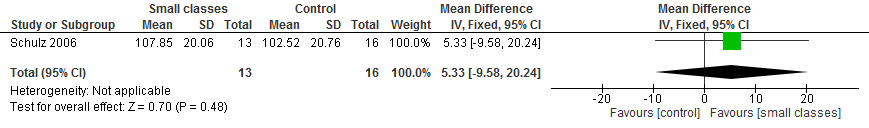


Comparison 5: Couple-focused classes versus standard care, outcome 5: Divorce/separation 5.5 years postnatal


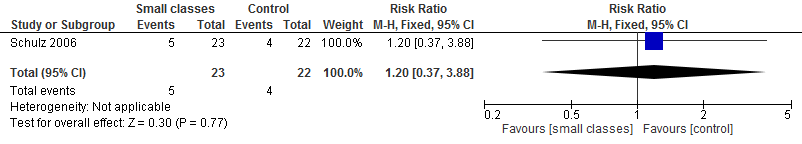


Comparison 6: Self-hypnosis versus standard care, outcome 1: Epidural analgesia


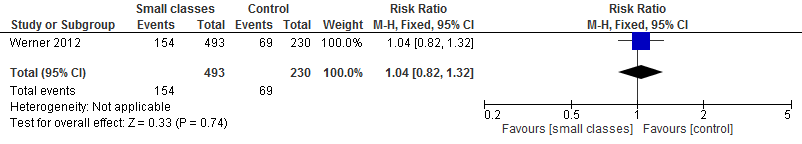


Comparison 6: Self-hypnosis versus standard care, outcome 2: Spontaneous delivery


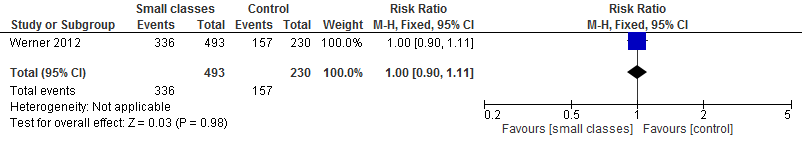


Comparison 6: Self-hypnosis versus standard care, outcome 3: Overall caesarean section


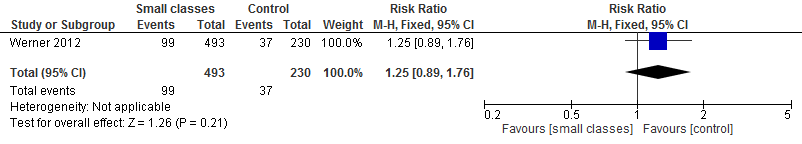


Comparison 6: Self-hypnosis versus standard care, outcome 4: Elective caesarean section


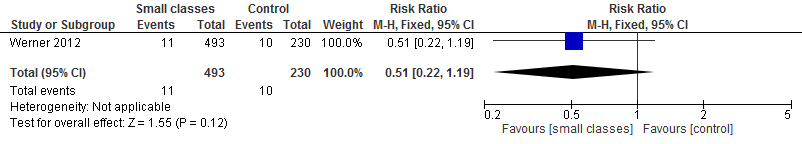


Comparison 6: Self-hypnosis versus standard care, outcome 5: Emergency caesarean section


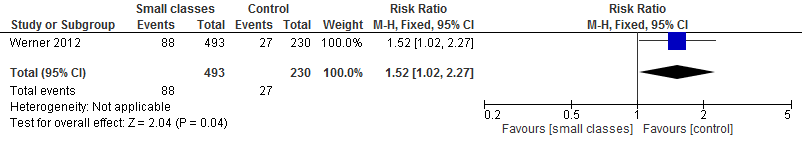


Comparison 6: Self-hypnosis versus standard care, outcome 6: Vacuum extraction


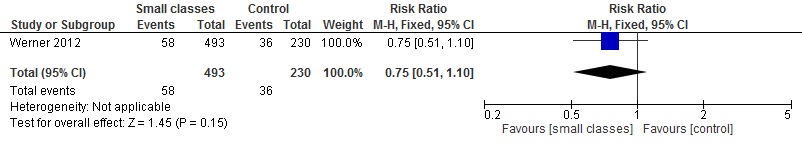


Comparison 6: Self-hypnosis versus standard care, outcome 7: Oxytocin augmentation


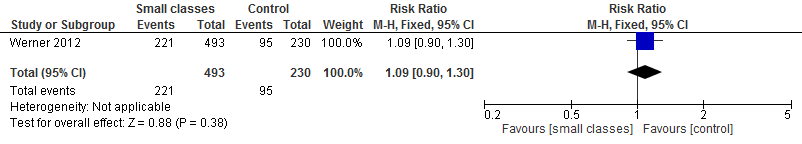


Comparison 6: Self-hypnosis versus standard care, outcome 8: Labor induction


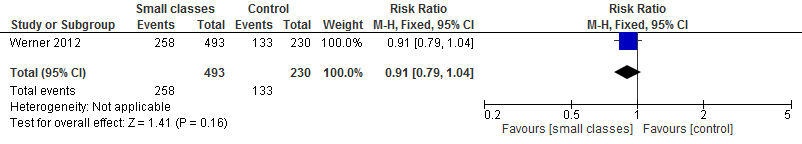


Comparison 6: Self-hypnosis versus standard care, outcome 9: Any breast feeding 4 months postnatal


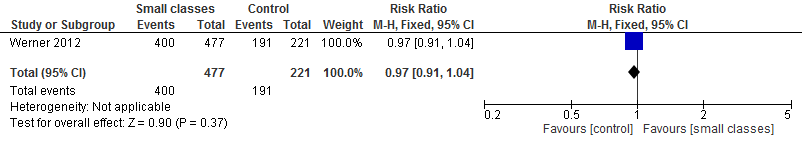


Comparison 7: General antenatal education classes versus standard care, outcome 1: Overall pain relief


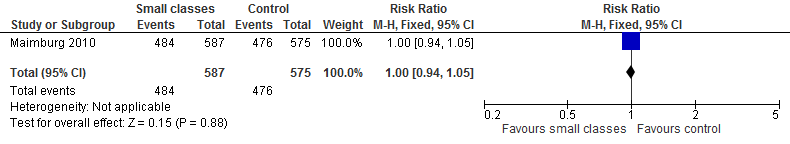


Comparison 7: General antenatal education classes versus standard care, outcome 2: Overall pharmacological pain relief


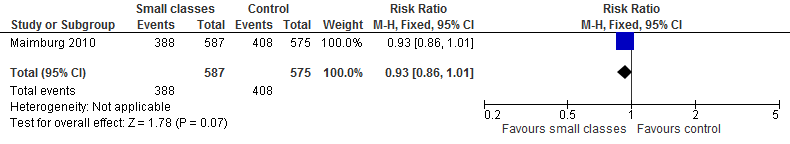


Comparison 7: General antenatal education classes versus standard care, outcome 3: Epidural analgesia


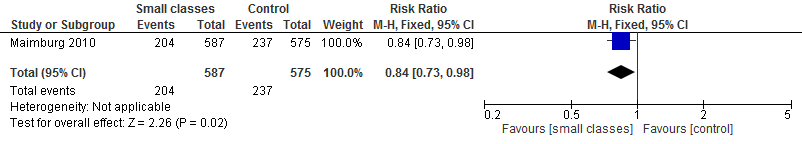


Comparison 7: General antenatal education classes versus standard care, outcome 4: Nitrous oxide/oxygen


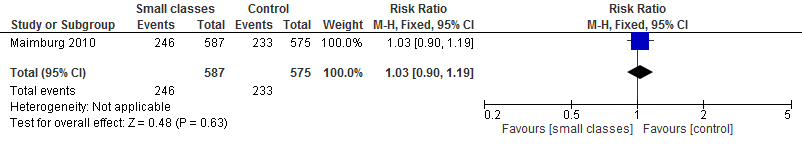


Comparison 7: General antenatal education classes versus standard care, outcome 5: Intramuscular morphine

**
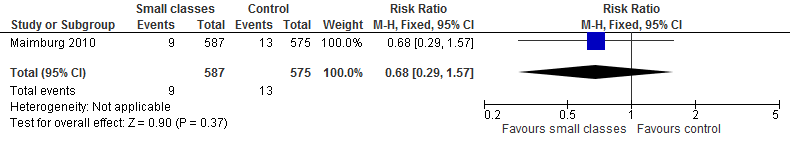
**

Comparison 7: General antenatal education classes versus standard care, outcome 6: Pudendal nerve block

**
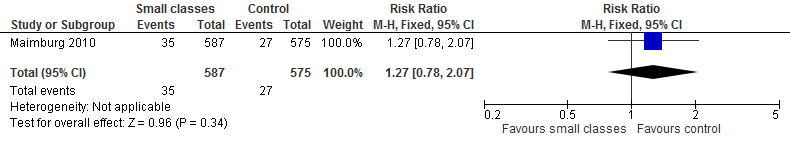
**

Comparison 7: General antenatal education classes versus standard care, outcome 7: Other pharmacological (primary halcion, codein, paracetamol)

**
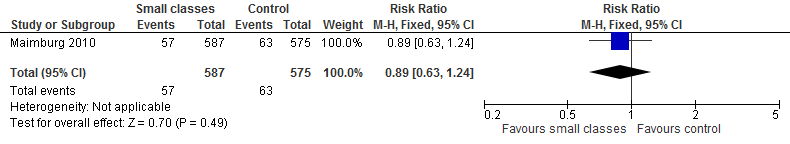
**

Comparison 7: General antenatal education classes versus standard care, outcome 8: Overall non-pharmacological pain relief

**
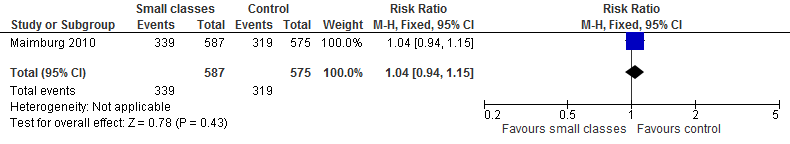
**

Comparison 7: General antenatal education classes versus standard care, outcome 9: Water immersion

**
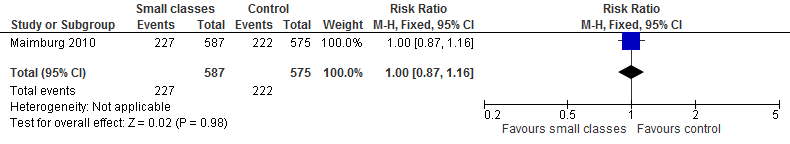
**

Comparison 7: General antenatal education classes versus standard care, outcome 10: Acupuncture


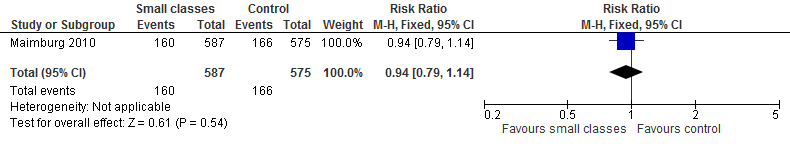


Comparison 7: General antenatal education classes versus standard care, outcome 11: Intracutaneous sterile water injection

**
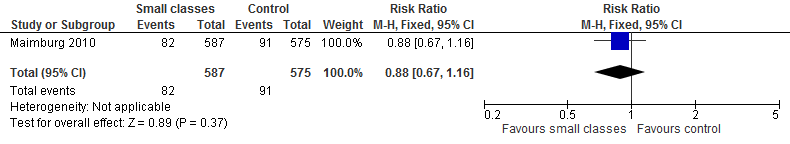
**

Comparison 7: General antenatal education classes versus standard care, outcome 12: Spontaneous delivery


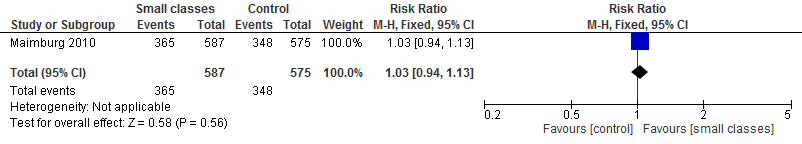


Comparison 7: General antenatal education classes versus standard care, outcome 13: Overall caesarean section


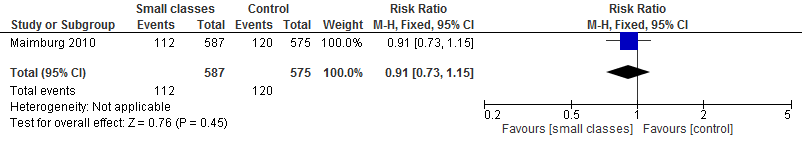


Comparison 7: General antenatal education classes versus standard care, outcome 14: Elective caesarean section


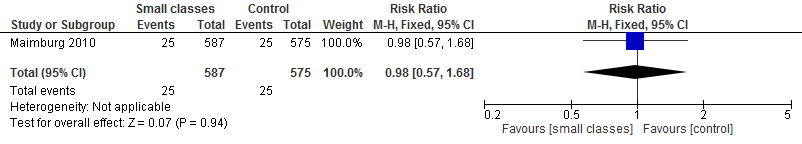


Comparison 7: General antenatal education classes versus standard care, outcome 15: Emergency caesarean section


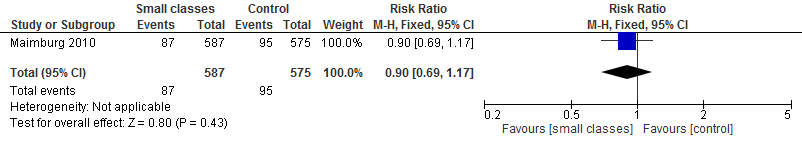


Comparison 7: General antenatal education classes versus standard care, outcome 16: Vacuum extraction


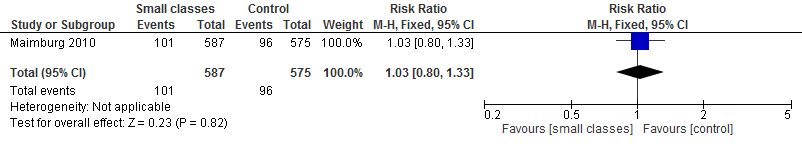


Comparison 7: General antenatal education classes versus standard care, outcome 17: Oxytocin augmentation


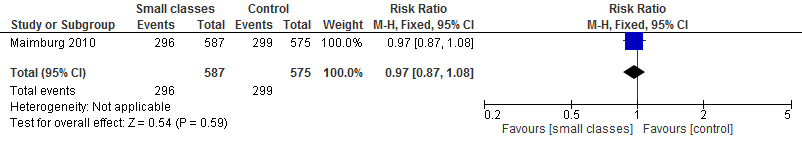


Comparison 7: General antenatal education classes versus standard care, outcome 18: Labor induction


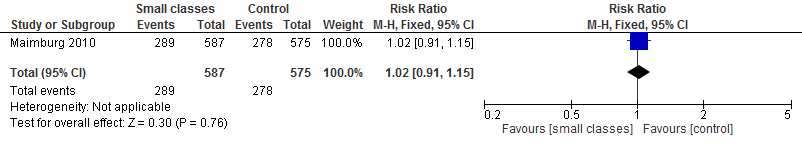


Comparison 7: General antenatal education classes versus standard care, outcome 19: Sufficient knowledge about breast feeding

**
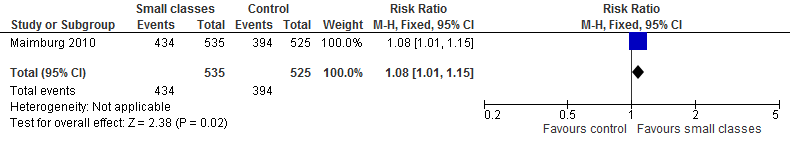
**

Comparison 7: General antenatal education classes versus standard care, outcome 20: Exclusive breast feeding 6 weeks postnatal


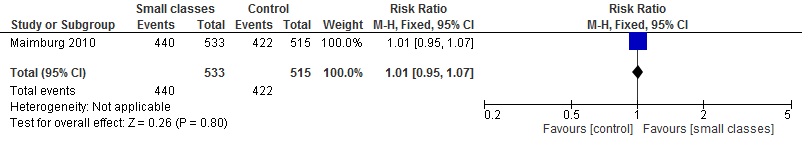


Comparison 7: General antenatal education classes versus standard care, outcome 21: Any breast feeding 6 weeks postnatal


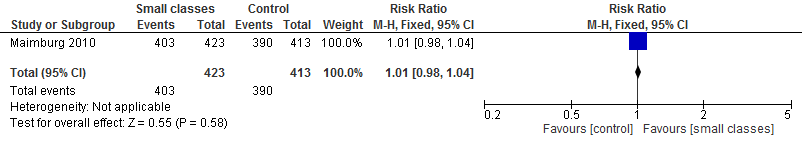


Comparison 7: General antenatal education classes versus standard care, outcome 22: Exclusive breast feeding 6 months postnatal


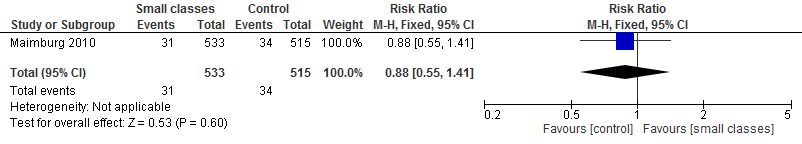


Comparison 7: General antenatal education classes versus standard care, outcome 23: Any breast feeding 6 months postnatal


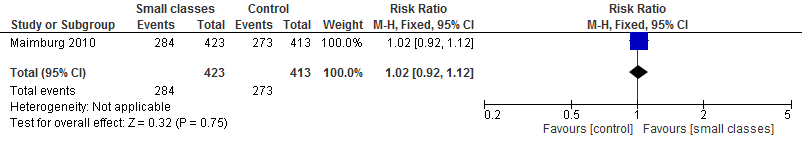


Comparison 7: General antenatal education classes versus standard care, outcome 24: Postnatal depression 6 weeks postnatal (EPDS)

**
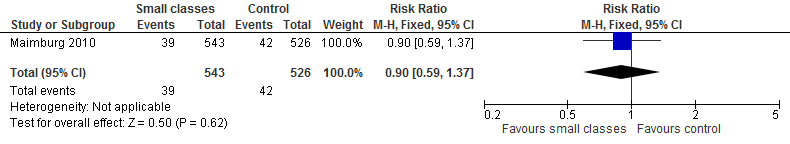
**

Comparison 7: General antenatal education classes versus standard care, outcome 25: Breast feeding self-efficacy 6 weeks postnatal


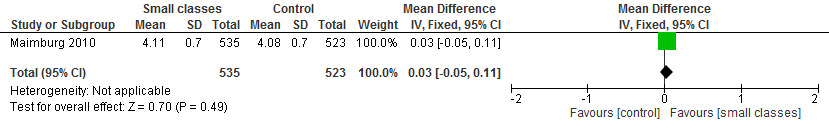


Comparison 8: Group prenatal care (20 hours) vs. individual prenatal care (2 hours), outcome 1: Prenatal and infant care knowledge


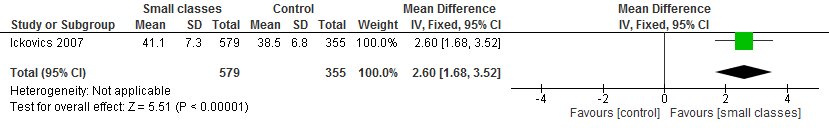


Comparison 8: Group prenatal care (20 hours) vs. individual prenatal care (2 hours), outcome 2: Readiness for labor and delivery


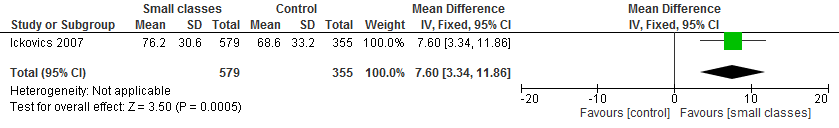


Comparison 8: Group prenatal care (20 hours) vs. individual prenatal care (2 hours), outcome 3: Readiness for infant care


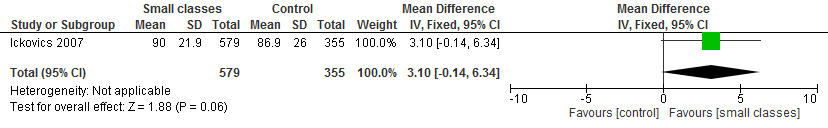


Comparison 8: Group prenatal care (20 hours) vs. individual prenatal care (2 hours), outcome 4: Prenatal distress


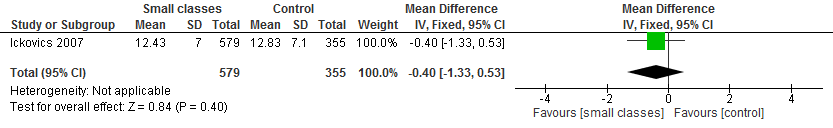


Comparison 9: Paternal education class versus standard care, outcome 1: Paternal knowledge


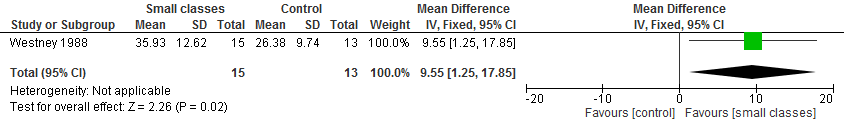


Comparison 9: Paternal education class versus standard care, outcome 2: Exclusive breast feeding 6 weeks postnatal


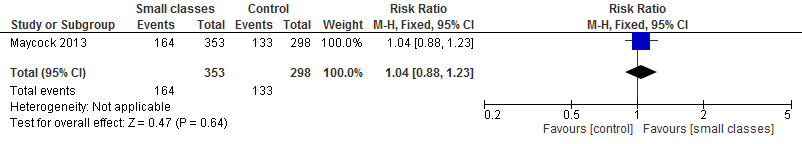


Comparison 9: Paternal education class versus standard care, outcome 3: Any breast feeding 6 weeks postnatal


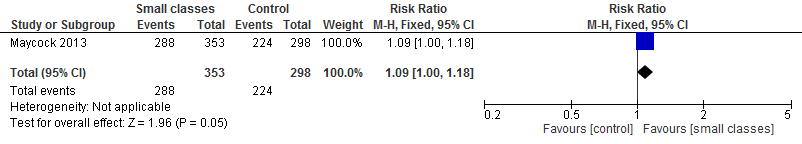


Comparison 10: Extra breast feeding sessions versus standard care, outcome 1: Spontaneous delivery


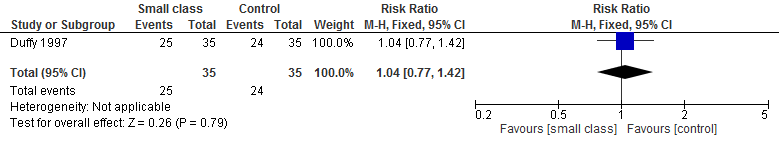


Comparison 10: Extra breast feeding sessions versus standard care, outcome 2: Overall caesarean section


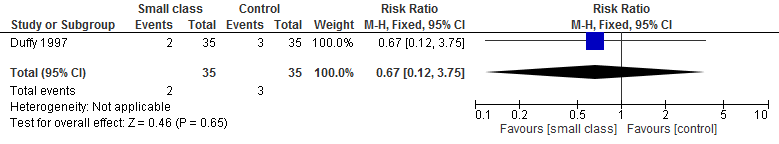


Comparison 10: Extra breast feeding sessions versus standard care, outcome 3: Vacuum extraction


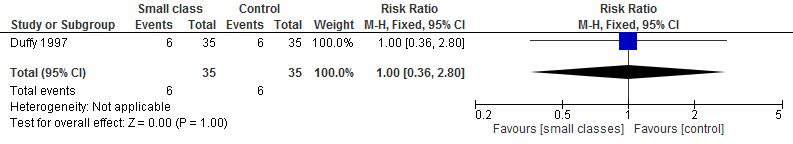


Comparison 10: Extra breast feeding sessions versus standard care, outcome 4: Forceps


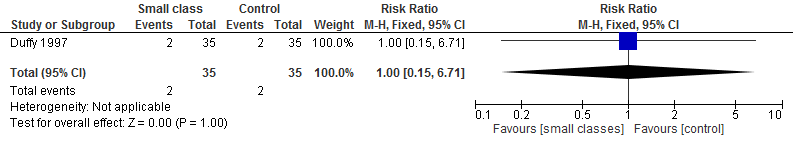


Comparison 10: Extra breast feeding sessions versus standard care, outcome 5: Breast feeding initiation – breast milk only

**
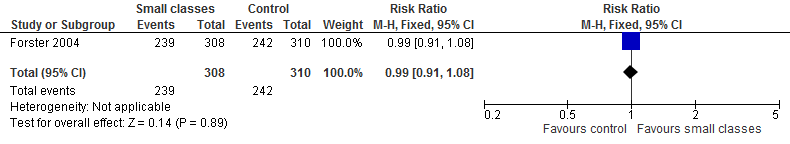
**

Comparison 10: Extra breast feeding sessions versus standard care, outcome 6: Breast feeding initiation – any breast milk


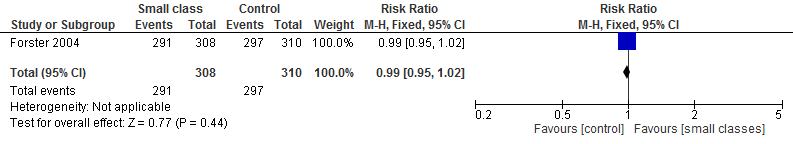


Comparison 10: Extra breast feeding sessions versus standard care, outcome 7: Exclusive breast feeding 6 weeks postnatal


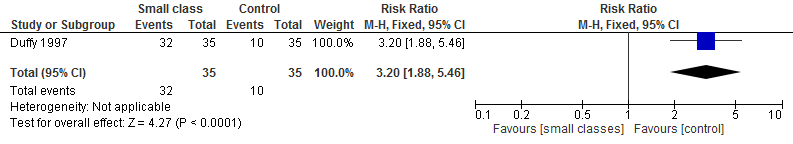


Comparison 10: Extra breast feeding sessions versus standard care, outcome 8: Breast milk only 8 weeks postnatal.

**
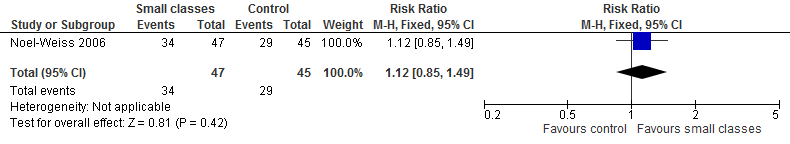
**

Comparison 10: Extra breast feeding sessions versus standard care, outcome 9: Any breast milk 8 weeks postnatal.

**
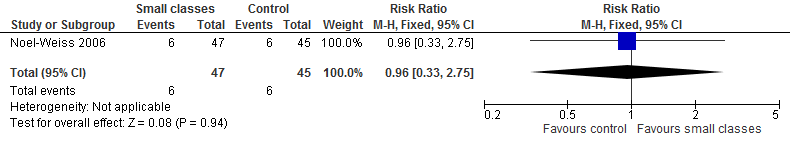
**

Comparison 10: Extra breast feeding sessions versus standard care, outcome 10: Exclusive breast feeding 6 months postnatal


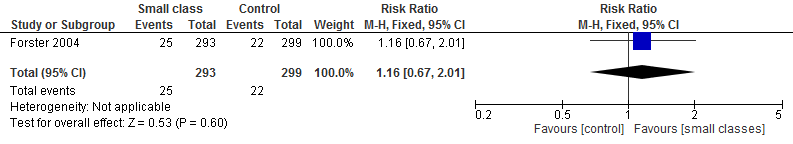


Comparison 10: Extra breast feeding sessions versus standard care, outcome 11: Breast milk only 6 months postnatal


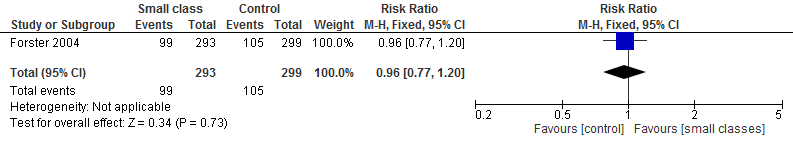


Comparison 10: Extra breast feeding sessions versus standard care, outcome 12: Any breast milk 6 months postnatal


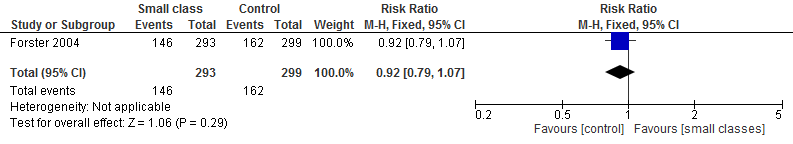


Comparison 10: Extra breast feeding sessions versus standard care, outcome 13: Breast feeding self-efficacy 4 weeks postnatal


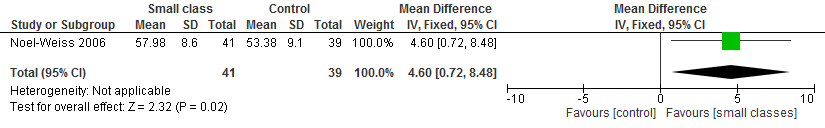


Comparison 10: Extra breast feeding sessions versus standard care, outcome 14: Breast feeding self-efficacy 8 weeks postnatal


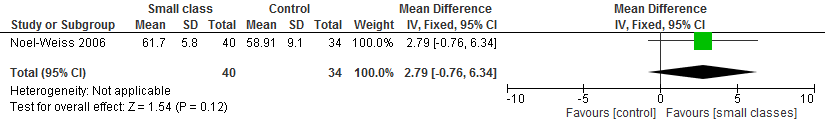


Comparison 11: Breast feeding classes versus one-to-one contact, outcome 1: Breast feeding initiation - one or more breast feedings per day

**
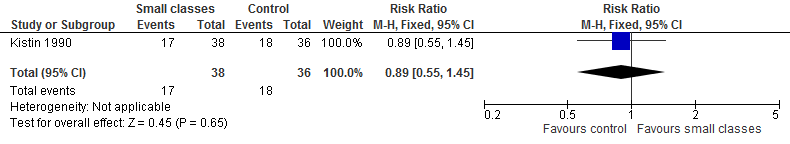
**

Comparison 11: Breast feeding classes versus one-to-one contact, outcome 2: Any breast feeding 12 weeks postnatal

**
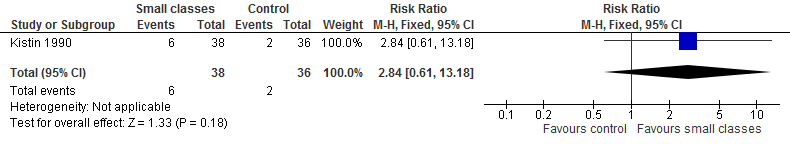
**

Comparison 12: Breast feeding classes versus breast feeding and childbirth pamphlets, outcome 1: Breast feeding initiation - main source of nutrition

**
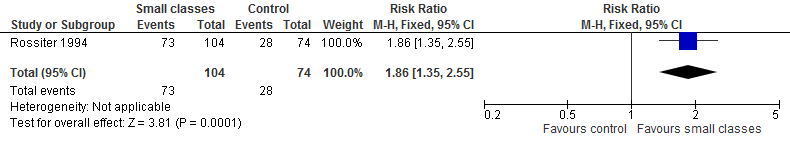
**

Comparison 12: Breast feeding classes versus breast feeding and childbirth pamphlets, outcome 2: Breast feeding as main source of nutrition 6 months postnatal

**
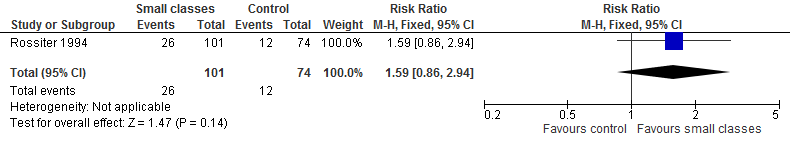
**
