# Supplementary material for: The effect of antenatal education in small classes on obstetric and psycho-social outcomes - a systematic review
Source: Syst Rev. 2015 Feb 28;4:20. doi: 10.1186/s13643-015-0010-x (PMC4355374; doi:10.1186/s13643-015-0010-x)
Supplement: Additional file 8: — Characteristics of excluded trials. The file contains a list of excluded trials with brief explanations of reasons for exclusion. [file 13643_2015_10_MOESM8_ESM.docx]

**Characteristics of excluded trials**

**Main reason for exclusion**

| **Not randomized trial** | |
| --- | --- |
| Elliot 2000 | Not a randomized controlled trial. |
| Turan 2003 | Not a randomized controlled trial. |
| **Outcome outside the scope of this review** | |
| Mendelson 2008 | Outcome: health behaviors, glycemic control, and neonatal outcomes among women with gestational diabetes. |
| Carter 1989 | Outcome: congenital toxoplasmosis. |
| Haugland 2006 | Outcome: pelvic girdle pain in pregnancy. |
| Morkved 2007 | Outcome: lumbopelvic pain, sick leave and functional status. |
| Guelinckx 2010 | Outcome: energy intake, physical activity, dietary habits and gestational weight gain in obese pregnant women. |
| Bogaerts 2013 | Outcome: gestational weight among obese pregnant women. |
| Stafne 2012 | Outcome: lumbopelvic pain. |
| Bonell 2013 | Outcome: teenage pregnancy. |
| Miquelutti 2013 | Outcome: lumbopelvic pain and urinary incontinence. |
| Hunter 2005 | Outcome: prenatal diagnosis. |
| Hui 2012 | Outcome: excessive gestational weight gain, physical activity, food intake. |
| **Experimental condition outside the scope of this review** | |
| Hunt 1976 | Experimental condition: nutrition education. |
| Shapiro 2011 | Experimental condition given as either a prenatal or postnatal workshop. More than half of the participants received the workshop postnatally. |
| Barakat 2008 | Experimental condition: exercise. |
| **Individual-based intervention** | |
| Halonen 1985 | Individual-based intervention aiming to reduce postpartum distress using relaxation training. |
| Leventhal 1989 | Individual-based intervention aiming to increase positive childbirth experience. |
| Subramanian 2012 | Individual-based intervention focusing on reducing behavioral and psychosocial risks. |
| Hayes 2001 | Individual-based intervention aiming to decrease postnatal depression. |
| Mcleod 2004 | Individual-based smoking and breast feeding intervention. |
| Kimber 2008 | Individual-based music and massage intervention delivered during birth. |
| Spinelli 2013 | Individual-based psychotherapy intervention focused on reducing postnatal depression. |
| **Same dose of antenatal education in experimental and control condition** | |
| Durham 1986 | Only difference between conditions was music as a conditioning aid in childbirth. |
| Coffman 1994 | Antenatal classes had different focus areas. Focus in experimental condition was partner support. |
| Wolfberg 2004 | Antenatal classes had different focus areas. Focus in the experimental condition was breast feeding support. |
| Timpano 2011 | Antenatal classes had different focus areas. The experimental condition was focused on OCD behavior – the control condition was focused more generally on anxiety. |
| Svensson 2009 | Antenatal classes had different focus areas. More focus on parenting issues in the experimental condition with the aim of improving parental coping. |
| Bergstrom 2009 | Antenatal classes had different focus areas. Focus in experimental condition was on natural birth and coping med labor pain. |
| Zimmermann-Tansella 1979 | Antenatal classes had different teaching methods. The experimental education was focus on body sensations and the control condition focus was on lectures and discussions. |
| Kozinszky 2012 | Antenatal classes had different focus areas. Focus in experimental condition was on psycho-education and psychotherapy for decreasing postpartum depression symptomatology. |
| Hawkins 2006 | Only difference between conditions was a booklet and video segments on relationship deterioration. |
| **Co-interventions in addition to antenatal education in small classes** | |
| Sciacca 1995 | Additional presents for breastfeeding, which was the main outcome. |
| Koniak-griffin 2000 | Additional 17 home visits aiming at increasing health and social outcomes, and mother-child interaction. |
| Klerman 2001 | Additional individual sessions several times during pregnancy. The intervention aimed at improving pregnancy outcomes and patients’ knowledge of risks, satisfaction with care and behavior. |
| Doherty 2006 | Additional home visits. The purpose of the intervention was to increase father involvement and skills with infants during the transition to parenthood. |
| Wambach 2011 | Additional home visits and telephone counselling. The intervention focused on breastfeeding support and education. |
| Kieffer 2013 | Additional home visits. The aim of the intervention was to reduce depressive symptoms among pregnant and early postpartum Latinas. |
| Halford 2010 | Additional home visits. The intervention aimed to promote a positive transition to parenthood. |
| Turan et al. 2001 | Additional individual telephone consultations. The paper is a summary of three studies on methods for including men in antenatal education. |
| **Inadequate information for analysis** | |
| Olenick 2010 | No description of control condition. The aim of the intervention was to improve breastfeeding outcomes. |
| Richter 2012 | No description of control condition. The aim of the intervention was to reduce stress in pregnant women in high risk of stress. |
| Wolfson 1992 | No information on the number of participants providing outcome data at each time point by group. This trial studied the effect of parent training on infant sleeping patters, parents’ stress, and perceived parental competence. |
| Lavender 2005 | No information on number of participants providing outcome data at each time point by group. This trial evaluated the effect of a breastfeeding intervention on breastfeeding duration. |
| Schachman 2004 | No standard deviations on means. The aim of the intervention was to increase prenatal and postpartum maternal role adaptation. |
| Midmer 1995 | No standard deviations on means. The aim of the intervention was among other things to increase marital adjustment, and postpartum adjustment. |
| Matthey 2004 | Results are presented as stratified analyses – no raw effect is presented. The aim of the intervention was to increase postpartum psychosocial adjustment. |
| Kermeen 1995 | Only F statistics and p-values for the comparison between groups are presented - no effect size measures. The aim of the intervention was to lower the potential negative effect of becoming a parent. |

Elliott SA, Leverton TJ, Sanjack M, Turner H, Cowmeadow P, Hopkins J, Bushnell D: **Promoting mental health after childbirth: a controlled trial of primary prevention of postnatal depression.** *The British journal of clinical psychology / the British Psychological Society* 2000, **39 ( Pt 3):**223-241.

Turan JM, Say L: **Community-based antenatal education in Istanbul, Turkey: effects on health behaviours.** *Health policy and planning* 2003, **18:**391-398.

Mendelson SG, McNeese-Smith D, Koniak-Griffin D, Nyamathi A, Lu MC: **A community-based parish nurse intervention program for Mexican American women with gestational diabetes.** *Journal of obstetric, gynecologic, and neonatal nursing : JOGNN / NAACOG* 2008, **37:**415-425.

Carter AO, Gelmon SB, Wells GA, Toepell AP: **The effectiveness of a prenatal education programme for the prevention of congenital toxoplasmosis.** *Epidemiology and infection* 1989, **103:**539-545.

Haugland KS, Rasmussen S, Daltveit AK: **Group intervention for women with pelvic girdle pain in pregnancy. A randomized controlled trial.** *Acta obstetricia et gynecologica Scandinavica* 2006, **85:**1320-1326.

Morkved S, Salvesen KA, Schei B, Lydersen S, Bo K: **Does group training during pregnancy prevent lumbopelvic pain? A randomized clinical trial.** *Acta obstetricia et gynecologica Scandinavica* 2007, **86:**276-282.

Guelinckx I, Devlieger R, Mullie P, Vansant G: **Effect of lifestyle intervention on dietary habits, physical activity, and gestational weight gain in obese pregnant women: a randomized controlled trial.** *The American journal of clinical nutrition* 2010, **91:**373-380.

Bogaerts AF, Devlieger R, Nuyts E, Witters I, Gyselaers W, Van den Bergh BR: **Effects of lifestyle intervention in obese pregnant women on gestational weight gain and mental health: a randomized controlled trial.** *International journal of obesity (2005)* 2013, **37:**814-821.

Stafne SN, Salvesen KA, Romundstad PR, Stuge B, Morkved S: **Does regular exercise during pregnancy influence lumbopelvic pain? A randomized controlled trial.** *Acta obstetricia et gynecologica Scandinavica* 2012, **91:**552-559.

Bonell C, Maisey R, Speight S, Purdon S, Keogh P, Wollny I, Sorhaindo A, Wellings K: **Randomized controlled trial of 'teens and toddlers': a teenage pregnancy prevention intervention combining youth development and voluntary service in a nursery.** *Journal of adolescence* 2013, **36:**859-870.

Miquelutti MA, Cecatti JG, Makuch MY: **Evaluation of a birth preparation program on lumbopelvic pain, urinary incontinence, anxiety and exercise: a randomized controlled trial.** *BMC pregnancy and childbirth* 2013, **13:**154.

Hunter AG, Cappelli M, Humphreys L, Allanson JE, Chiu TT, Peeters C, Moher D, Zimak A: **A randomized trial comparing alternative approaches to prenatal diagnosis counseling in advanced maternal age patients.** *Clinical genetics* 2005, **67:**303-313.

Hui A, Back L, Ludwig S, Gardiner P, Sevenhuysen G, Dean H, Sellers E, McGavock J, Morris M, Bruce S, et al: **Lifestyle intervention on diet and exercise reduced excessive gestational weight gain in pregnant women under a randomised controlled trial.** *BJOG : an international journal of obstetrics and gynaecology* 2012, **119:**70-77.

Hunt IF, Jacob M, Ostegard NJ, Masri G, Clark VA, Coulson AH: **Effect of nutrition education on the nutritional status of low-income pregnant women of Mexican descent.** *The American journal of clinical nutrition* 1976, **29:**675-684.

Shapiro AF, Nahm EY, Gottman JM, Content K: **Bringing baby home together: examining the impact of a couple-focused intervention on the dynamics within family play.** *The American journal of orthopsychiatry* 2011, **81:**337-350.

Barakat R, Stirling JR, Lucia A: **Does exercise training during pregnancy affect gestational age? A randomised controlled trial.** *British journal of sports medicine* 2008, **42:**674-678.

Halonen JS, Passman RH: **Relaxation training and expectation in the treatment of postpartum distress.** *Journal of consulting and clinical psychology* 1985, **53:**839-845.

Leventhal EA, Leventhal H, Shacham S, Easterling DV: **Active coping reduces reports of pain from childbirth.** *Journal of consulting and clinical psychology* 1989, **57:**365-371.

Subramanian S, Katz KS, Rodan M, Gantz MG, El-Khorazaty NM, Johnson A, Joseph J: **An integrated randomized intervention to reduce behavioral and psychosocial risks: pregnancy and neonatal outcomes.** *Maternal and child health journal* 2012, **16:**545-554.

Hayes BA, Muller R, Bradley BS: **Perinatal depression: a randomized controlled trial of an antenatal education intervention for primiparas.** *Birth (Berkeley, Calif)* 2001, **28:**28-35.

McLeod D, Pullon S, Benn C, Cookson T, Dowell A, Viccars A, White S, Green R, Crooke M: **Can support and education for smoking cessation and reduction be provided effectively by midwives within primary maternity care?** *Midwifery* 2004, **20:**37-50.

Kimber L, McNabb M, Mc Court C, Haines A, Brocklehurst P: **Massage or music for pain relief in labour: a pilot randomised placebo controlled trial.** *European journal of pain (London, England)* 2008, **12:**961-969.

Spinelli MG, Endicott J, Leon AC, Goetz RR, Kalish RB, Brustman LE, Carmona YR, Meyreles Q, Vega M, Schulick JL: **A controlled clinical treatment trial of interpersonal psychotherapy for depressed pregnant women at 3 New York City sites.** *The Journal of clinical psychiatry* 2013, **74:**393-399.

Durham L, Collins M: **The effect of music as a conditioning aid in prepared childbirth education.** *Journal of obstetric, gynecologic, and neonatal nursing : JOGNN / NAACOG* 1986, **15:**268-270.

Coffman S, Levitt MJ, Brown L: **Effects of clarification of support expectations in prenatal couples.** *Nursing research* 1994, **43:**111-116.

Wolfberg AJ, Michels KB, Shields W, O'Campo P, Bronner Y, Bienstock J: **Dads as breastfeeding advocates: results from a randomized controlled trial of an educational intervention.** *American journal of obstetrics and gynecology* 2004, **191:**708-712.

Timpano KR, Abramowitz JS, Mahaffey BL, Mitchell MA, Schmidt NB: **Efficacy of a prevention program for postpartum obsessive-compulsive symptoms.** *Journal of psychiatric research* 2011, **45:**1511-1517.

Svensson J, Barclay L, Cooke M: **Randomised-controlled trial of two antenatal education programmes.** *Midwifery* 2009, **25:**114-125.

Bergstrom M, Kieler H, Waldenstrom U: **Effects of natural childbirth preparation versus standard antenatal education on epidural rates, experience of childbirth and parental stress in mothers and fathers: a randomised controlled multicentre trial.** *BJOG : an international journal of obstetrics and gynaecology* 2009, **116:**1167-1176.

Zimmermann-Tansella C, Dolcetta G, Azzini V, Zacche G, Bertagni P, Siani R, Tansella M: **Preparation courses for childbirth in primipara. A comparison.** *Journal of psychosomatic research* 1979, **23:**227-233.

Kozinszky Z, Dudas RB, Devosa I, Csatordai S, Toth E, Szabo D, Sikovanyecz J, Barabas K, Pal A: **Can a brief antepartum preventive group intervention help reduce postpartum depressive symptomatology?** *Psychotherapy and psychosomatics* 2012, **81:**98-107.

Hawkins AJ, Fawcett EB, Carroll JS, Gilliland TT: **The marriage moments program for couples transitioning to parenthood: divergent conclusions from formative and outcome evaluation data.** *Journal of family psychology : JFP : journal of the Division of Family Psychology of the American Psychological Association (Division 43)* 2006, **20:**561-570.

Sciacca JP, Phipps BL, Dube DA, Ratliff MI: **Influences on breast-feeding by lower-income women: an incentive-based, partner-supported educational program.** *Journal of the American Dietetic Association* 1995, **95:**323-328.

Koniak-Griffin D, Anderson NL, Verzemnieks I, Brecht ML: **A public health nursing early intervention program for adolescent mothers: outcomes from pregnancy through 6 weeks postpartum.** *Nursing research* 2000, **49:**130-138.

Klerman LV, Ramey SL, Goldenberg RL, Marbury S, Hou J, Cliver SP: **A randomized trial of augmented prenatal care for multiple-risk, Medicaid-eligible African American women.** *American journal of public health* 2001, **91:**105-111.

Doherty WJ, Erickson MF, LaRossa R: **An intervention to increase father involvement and skills with infants during the transition to parenthood.** *Journal of family psychology : JFP : journal of the Division of Family Psychology of the American Psychological Association (Division 43)* 2006, **20:**438-447.

Wambach KA, Aaronson L, Breedlove G, Domian EW, Rojjanasrirat W, Yeh HW: **A randomized controlled trial of breastfeeding support and education for adolescent mothers.** *Western journal of nursing research* 2011, **33:**486-505.

Kieffer EC, Caldwell CH, Welmerink DB, Welch KB, Sinco BR, Guzman JR: **Effect of the healthy MOMs lifestyle intervention on reducing depressive symptoms among pregnant Latinas.** *American journal of community psychology* 2013, **51:**76-89.

Halford WK, Petch J, Creedy DK: **Promoting a positive transition to parenthood: a randomized clinical trial of couple relationship education.** *Prevention science : the official journal of the Society for Prevention Research* 2010, **11:**89-100.

Turan JM, Nalbant H, Bulut A, Sahip Y: **Including expectant fathers in antenatal education programmes in Istanbul, Turkey.** *Reproductive health matters* 2001, **9:**114-125.

Olenick PL: **The Effect of Structured Group Prenatal Education on Breastfeeding Confidence, Duration, and Exclusivity to 12 Weeks Postpartum.** *Journal of Obstetric, Gynecologic, & Neonatal Nursing* 2010, **39:**S104-S105.

Richter J, Bittner A, Petrowski K, Junge-Hoffmeister J, Bergmann S, Joraschky P, Weidner K: **Effects of an early intervention on perceived stress and diurnal cortisol in pregnant women with elevated stress, anxiety, and depressive symptomatology.** *Journal of psychosomatic obstetrics and gynaecology* 2012, **33:**162-170.

Wolfson A, Lacks P, Futterman A: **Effects of parent training on infant sleeping patterns, parents' stress, and perceived parental competence.** *Journal of consulting and clinical psychology* 1992, **60:**41-48.

Lavender T, Baker L, Smyth R, Collins S, Spofforth A, Dey P: **Breastfeeding expectations versus reality: a cluster randomised controlled trial.** *BJOG : an international journal of obstetrics and gynaecology* 2005, **112:**1047-1053.

Schachman KA, Lee RK, Lederma RP: **Baby boot camp: facilitating maternal role adaptation among military wives.** *Nursing research* 2004, **53:**107-115.

Midmer D, Wilson L, Cummings S: **A randomized, controlled trial of the influence of prenatal parenting education on postpartum anxiety and marital adjustment.** *Family medicine* 1995, **27:**200-205.

Matthey S, Kavanagh DJ, Howie P, Barnett B, Charles M: **Prevention of postnatal distress or depression: an evaluation of an intervention at preparation for parenthood classes.** *Journal of affective disorders* 2004, **79:**113-126.

Kermeen P: **Improving postpartum marital relationships.** *Psychological reports* 1995, **76:**831-834.
